# A systematic review of trucking food, physical activity, and tobacco environments and tractor-trailer drivers’ related patterns and practices in the United States and Canada, 1993–2021

**DOI:** 10.1016/j.pmedr.2022.101760

**Published:** 2022-03-08

**Authors:** Bailey Houghtaling, Laura Balis, Leia Minaker, Khawlah Kheshaifaty, Randa Morgan, Carmen Byker Shanks

**Affiliations:** a School of Nutrition and Food Sciences, Louisiana State University (LSU) and LSU Agricultural Center, Baton Rouge, LA 70803, USA; b Pacific Institute for Research and Evaluation, Louisville Center, 401 W. Main St., Suite 2100, Louisville, KY 40202, USA; c School of Planning, University of Waterloo, Waterloo, ON, Canada; d Louisiana State University (LSU) Libraries, LSU, Baton Rouge, LA 70803, USA; e Gretchen Swanson Center for Nutrition, Omaha, NE 68114, USA; f Department of Health and Human Development, Montana State University, Bozeman, MT 59717, USA

**Keywords:** PA, Physical activity, U.S., United States, SNAP, Supplemental Nutrition Assistance Program, NEMS, Nutrition Environment Measures Survey, NEMS-V, Nutrition Environment Measures Survey-Vending, HEATWAI, The Healthy Trucking Worksites Audit Instrument, HEATS, Healthy Trucker Survey, HPLP II, The Health-Promoting Lifestyle Profile II, NAS, Nutrition Attitude Survey, FCI, Food Choices Index, MET, Metabolic equivalent tasks, BMI, Body Mass Index, CI, Confidence Interval, Kcal, Kilocalorie, SE, Standard error, Food environment, Physical activity environment, Tobacco, Truck drivers, Occupational health

## Abstract

Truckers in the United States (U.S.) and Canada are at high risk for noncommunicable disease. Although trucking built environments have been highlighted for intervention, no systematic review has assessed aspects of trucking environments that may influence food, physical activity (PA), and smoking patterns/practices. The purpose of this systematic review was to characterize the state of the science on trucking food, PA, and tobacco environments and to examine truckers’ food, PA, and tobacco patterns/practices. The Preferred Reporting Items for Systematic Reviews and Meta-Analyses guidelines were used. Five databases were selected for searching in April 2020 and 2021 using key terms constructed by a librarian. Peer-reviewed research with data about U.S. and Canadian truckers’ (i.e., drivers operating 18-wheelers or tractor trailers) food, PA, and/or tobacco environments and related patterns/practices were included. Quality was assessed using the Mixed Method Appraisal Tool. Thirty-eight studies were identified. Results included data from at least 16,600 truckers and 282 trucking settings in the U.S. (n = 32) and Canada (n = 6). Most studies were classified as quantitative descriptive and of poor quality (average score 3 of 7). The few studies (n = 4) that measured trucking food and PA environments characterized trucking sites as poor. Fifteen (47%) presented data about truckers’ perceptions of food or PA environments and highlighted prominent environmental barriers. Truckers’ food, PA, and smoking patterns and practices suggested poor diet quality, sedentary practices, and a high prevalence of smoking. The science of trucking food, PA, and tobacco environments is underdeveloped and requires much more focus using validated measures.

## Highlights

• The built environment is recognized to influence health patterns and practices.
• No review has explored trucking food, physical activity, and tobacco environments.
• Trucking built environment science is underdeveloped and requires validated tools.
• Future research should explore truckers’ views on built environment interventions.
• More emphasis on the trucking built environment and health equity is warranted.

## 1 Background

There are more than 2.1 million long-haul heavy and tractor-trailer drivers (herein ‘truckers’) traveling between 2,000 to 3,000 miles each week to haul freight (AllTrucking.com, 2016) in the United States (U.S.) and Canada (U.S. Bureau of Labor Statistics, 2019, Gill and Macdonald, 2013). Truckers can be on the road for two consecutive nights to several continuous weeks and rely on truck terminals, warehouses, truck stops, highway rest areas, and truck cabs (Hill et al., 2015) for daily needs due to time and tractor-trailer parking constraints (Hege et al., 2017, Apostolopoulos et al., 2013, Crizzle et al., 2020). Trucking populations are two times more likely to be medically classified as having obesity [kg(weight)/m(height)^2^ ≥ 30] than non-trucking populations and to report food, physical activity (PA), and tobacco patterns/practices misaligned with public health guidance (Health Canada, 2019, U.S. Department of Agriculture, 2020, U.S. Department of Health and Human Services, 2018). However, trucking-focused interventions have primarily focused on individual determinants of health (Ng et al., 2015), which are approaches less likely to show long-term and large-scale effectiveness amid unsupportive environments (Frieden, 2010).

While the need to transform built environments to promote truckers’ health is not a new concept (Ng et al., 2015, Crizzle et al., 2017), no systematic reviews have assessed aspects of trucking environments that may influence diet, PA, and tobacco use in relation to public health guidelines. For example, dietary patterns with adequate amounts of fruits and vegetables and limited saturated and *trans*-fat, added sugars, and sodium–nutrients typically found in ultra-processed products such as confections, sodas, and packaged meals–are recommended for the prevention of noncommunicable diseases in both the U.S. and Canadian dietary guidelines (Health Canada, 2019, U.S. Department of Agriculture, 2020). Additionally, engaging in 150 minutes of moderate-intensity aerobic PA with two days of strength training each week (HHS, 2018) and entirely refraining from using tobacco products is suggested (Schane et al., 2010). This gap is notable given environmental aspects like availability, affordability, marketing, and/or safety, are well recognized to influence population food (Glanz et al., 2005, Story et al., 2008, Swinburn et al., 2011), PA (Kahn et al., 2002, McCormack and Shiell, 2011, Sallis et al., 2012), and tobacco (Robertson et al., 2015, Paynter and Edwards, 2009) patterns and practices.

Using the terminology “patterns and practices” in place of “behaviors” acknowledges the structural forces that influence health-related choices to emphasize the need for solutions that target up-stream factors (Olstad and Kirkpatrick, 2021). This framing seems especially relevant for truckers who may be disproportionately exposed to less healthy products and spaces and at the same time may have limited resources to overcome salient, unhealthy environments due to high levels of stress and poor sleep habits as a condition of their work (Cohen and Babey, 2012, Apostolopoulos et al., 2014). Regarding the food environment, food-away-from-home typically has higher amounts of saturated fat, added sugars, and sodium than food prepared for home consumption (Cooksey-Stowers et al., 2017, Saksena et al., 2018) and “food swamps” or areas with high concentration of convenience/corner stores and restaurants that offer less healthy foods and beverages have been linked with negative health outcomes (Cooksey-Stowers et al., 2017, Houghtaling et al., 2021) and may characterize trucking settings (Hill et al., 2015, Apostolopoulos et al., 2012). Similarly, PA has been linked to infrastructure availability, safety, and connectedness (e.g., sidewalks, bike lanes) (Kahn et al., 2002, Heath et al., 2006) indicating lighting, traffic, and available amenities at truck and travel stops likely influence truckers’ PA patterns and practices. Last, point-of-sale strategies are one of the most dominant forms of current tobacco marketing (Lee et al., 2015, Ribisl et al., 2017) and exposure to these types of promotions has been associated with increased risk of smoking, impulse tobacco purchasing, and urges to buy tobacco (Robertson et al., 2015).

There is a need to examine the state of the science regarding trucking food, PA, and tobacco environments and truckers’ related patterns and practices to inform future research needs and policy, systems, and environmental change efforts to improve truckers’ workplace environments while on the road. Therefore, the purpose of the present systematic review was twofold: 1) to characterize the state of the science on trucking food, PA, and tobacco environments using data from both objective and subjective investigations; and 2) to examine truckers’ food, PA, and tobacco patterns/practices as initial evidence of truckers’ interaction with their environments.

## 2 Methods

The 2009 and 2020 Preferred Reporting Items for Systematic Reviews and Meta-Analyses (PRISMA) guidelines were followed as appropriate, given the review was initiated prior to the publication of the 2020 PRISMA version (Page et al., 2020, Moher et al., 2009). A protocol was pre-registered on Open Science Framework (doi https://doi.org/10.17605/OSF.IO/JRMHA). Review members are U.S. and Canadian public health scholars with an emphasis on social and built environment determinants of health, as well as a library partner with reviewing expertise. Theory used to inform the present review included the Social-Ecological Model (Glanz et al., 2008) and Social Cognitive Theory (Glanz et al., 2008) in recognition of environmental influences on health patterns/practices and the reciprocal nature of human-environment interactions.

### 2.1 Search strategy

Key terms were developed in collaboration with the librarian (RM) and focused on population (e.g., truck*, “long-haul truck drivers”), location (i.e., U.S. states and Canadian provinces), health patterns/practices (e.g., diet*, exercise*, tobacco), and settings (e.g., “truck stop”, workplace*). The complete search strategy is shown in supplementary file I. Five databases (Medline, PubMed, Web of Science, CINHAL, and APA PsychInfo) were selected for systematic searching in April of 2020 and 2021. Medline, CINHAL Complete, and APA PsychInfo are hosted on the EBSCOhost platform. Medline’s coverage ranges from 1950 to present, CINAHL Complete coverage ranges from the mid-1930’s to current, and APA PsychInfo coverage dates back to the 17th century. PubMed is hosted through the U.S. National Library of Medicine with coverage ranging from the 1940s to present day. The Web of Science Core Collection, hosted through Clarivate Analytics, ranges from 1900 to the present. The lead author completed database searching and extracted all results to EndNote X9. Two authors (BH and KK) reviewed source titles and abstracts independently and reached a consensus regarding those requiring full-text review. The full-text reviewing was also conducted independently among the two authors with consensus reached for review inclusion. Ongoing Google searches and a review of source references were also used to identify articles for review inclusion.

#### 2.1.1 Inclusion criteria

Populations operating 18-wheelers or tractor-trailers were considered ‘truckers’ in this research. Peer-reviewed, original research published in English was a requirement. No date limitations were used. Sources needed to have data about food, PA, and/or tobacco patterns and practices and/or food, PA, and/or tobacco environments in settings accessed by U.S. or Canadian truckers while on the road, such as truck terminals, warehouses, truck stops, highway rest areas, and truck cabs (Hill et al., 2015). The decision to focus only on research conducted in the U.S. and Canada was determined by co-author expertise regarding food, PA, and tobacco policy, systems, and environmental research in both the U.S. (BH, LB, CBS) and Canada (LM). Both objective (e.g., results of an environmental assessment) and subjective (e.g., truckers’ perceptions of environments) data were of interest. Baseline data from intervention studies were included, when applicable. “Food” included alcohol (a non-essential macronutrient with seven kilocalories per gram) and other beverages (e.g., coffee, water, sugar-sweetened beverages).

#### 2.1.2 Exclusion criteria

Sources did not meet review scope if: 1) in a language other than English; 2) not peer-reviewed (e.g., gray literature) or original research (e.g., commentaries); 3) among populations not located in the U.S. or Canada; 4) among non-trucking populations as defined in this research, such as bus or local parcel delivery drivers; and 5) not including results about food, PA, and/or tobacco environments or patterns/practices (e.g., body mass index, chronic disease diagnosis, marijuana use).

#### 2.1.3 Review outcomes

Microsoft® Excel® (2018 Microsoft Corporation, Redmond, WA, USA) was used to guide data extraction for article characteristics (study objective, design, location), environmental data (setting, measurement, and food, PA, and/or tobacco environment results), and data about related perceptions and patterns/practices (recruitment strategy, participant characteristics, qualitative or quantitative measures, and results). Categories for data extraction are presented in the below Tables. A graduate research assistant with a background in community health extracted data from all sources (KK) and co-authors (BH, LB, LM, and CBS) each reviewed extractions for suggested changes. A consensus was reached between two co-authors for all data points. Three topic experts (BH, LB, and LM) constructed a narration of results by topic area for food, PA, and tobacco, respectively, using evidence tables.

#### 2.1.4 Study quality

Research quality was assessed independently between two authors (BH and LB) using the 2018 Mixed-Method Appraisal Tool (Hong et al., 2019, Souto et al., 2015). This tool allows for the assessment of quality among quantitative, qualitative, and mixed-method studies (Pluye and Hong, 2014), which were all captured in the review scope. For each study type, the 2018 Mixed-Method Appraisal Tool prompts seven “Yes”, “No”, or “Can’t Tell” responses to questions about study rationale, design, methods, and conclusions (Hong et al., 2019, Souto et al., 2015). Discrepancies in ratings were discussed until a consensus was reached. Quality ratings are communicated below based on the number of “Yes” determinations regarding study quality indices.

## 3 Results

**Fig. 1:**
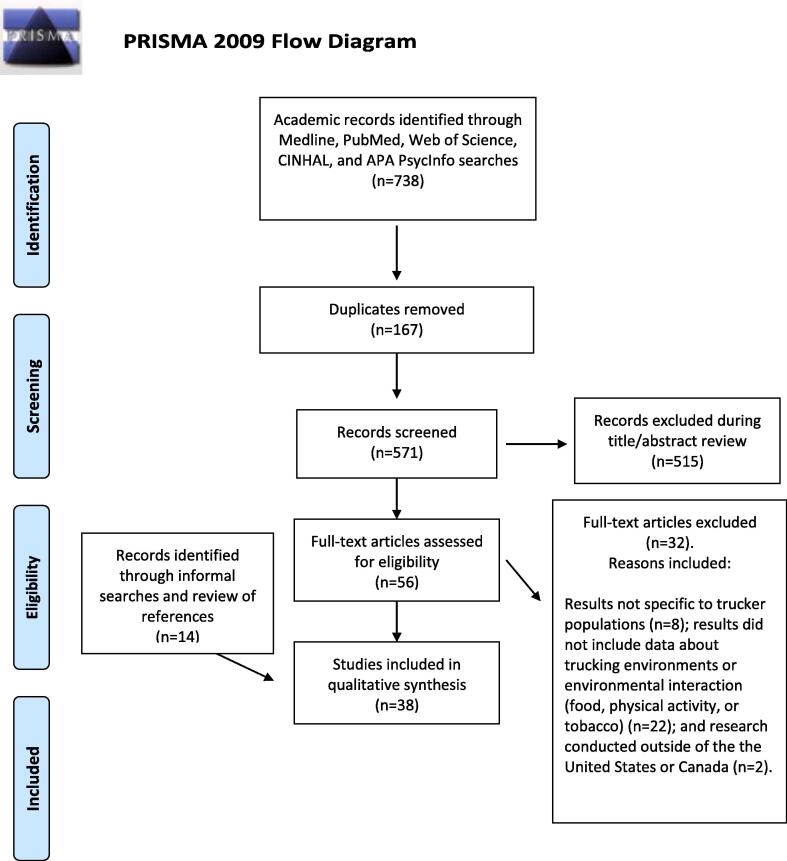
PRISMA Flow Diagram.

Thirty-eight original research articles published between the years 1993 and 2021 were identified (Apostolopoulos et al., 2013, Crizzle et al., 2020, Apostolopoulos et al., 2012, Angeles et al., 2014, Apostolopoulos et al., 2016, Apostolopoulos et al., 2011, Bachmann et al., 2018, Birdsey et al., 2015, Van Hemel and Rogers, 1998, Heaton and Griffin, 2015, Hege et al., 2019, Holmes and Power, 1996, Korelitz et al., 1993, Layne et al., 2009, Lemke et al., 2016, Lincoln et al., 2018, McDonough et al., 2014, McGuirt et al., 2019, Mullins et al., 2013, Olson et al., 2009, Olson et al., 2016, Olson et al., 2016, Passey et al., 2014, Ronna et al., 2016, Sieber et al., 2014, Staško and Neale, 2007, Thiese et al., 2015, Turner and Reed, 2011, Versteeg et al., 2018, Wenger, 2008, Whitfield Jacobson et al., 2007, Gay Anderson and Riley, 2008, Crizzle et al., 2020, Johnson et al., 2021, Shattell et al., 2012, Shattell et al., 2012, Solomon et al., 2004, Williams et al., 2017). The 2009 PRISMA diagram is shown in Fig. 1. Most sources were classified as quantitative descriptive research, including baseline data included from intervention research (n = 27; 71%) (Apostolopoulos et al., 2013, Apostolopoulos et al., 2012, Lincoln et al., 2018, Ronna et al., 2016, Sieber et al., 2014, Thiese et al., 2015, Turner and Reed, 2011, Whitfield Jacobson et al., 2007, Crizzle et al., 2020, Shattell et al., 2012, Solomon et al., 2004, Angeles et al., 2014, Apostolopoulos et al., 2016, Apostolopoulos et al., 2011, Bachmann et al., 2018, Birdsey et al., 2015, Van Hemel and Rogers, 1998, Heaton and Griffin, 2015, Hege et al., 2019, Holmes and Power, 1996, Korelitz et al., 1993, Layne et al., 2009, McGuirt et al., 2019, Mullins et al., 2013, Olson et al., 2009, Olson et al., 2016, Olson et al., 2016). Data from at least 16,600 truckers and 282 unique trucking settings in the U.S. (n = 32 studies; 84%) (Apostolopoulos et al., 2013, Apostolopoulos et al., 2012, Apostolopoulos et al., 2016, Apostolopoulos et al., 2011, Bachmann et al., 2018, Birdsey et al., 2015, Van Hemel and Rogers, 1998, Heaton and Griffin, 2015, Hege et al., 2019, Holmes and Power, 1996, Korelitz et al., 1993, Layne et al., 2009, Lemke et al., 2016, Lincoln et al., 2018, McGuirt et al., 2019, Mullins et al., 2013, Olson et al., 2009, Olson et al., 2016, Olson et al., 2016, Passey et al., 2014, Ronna et al., 2016, Sieber et al., 2014, Staško and Neale, 2007, Thiese et al., 2015, Turner and Reed, 2011, Wenger, 2008, Whitfield Jacobson et al., 2007, Gay Anderson and Riley, 2008, Shattell et al., 2012, Shattell et al., 2012, Solomon et al., 2004, Williams et al., 2017) and Canada (n = 6 studies; 16%) (Crizzle et al., 2020, Angeles et al., 2014, McDonough et al., 2014, Versteeg et al., 2018, Crizzle et al., 2020, Johnson et al., 2021) were synthesized in this review. Study characteristics of included studies are shown in Table 1. Using the Mixed Method Appraisal Tool (Hong et al., 2019, Souto et al., 2015), studies scored on average 3 and ranged from 0 to 7 (possible range of 0–7; with 0 used to indicate lowest and 7 highest quality). Quality review decisions are available in the supplementary file II.

**Table 1:** Original Research Included in the Systematic Review of Trucking Food, Physical Activity (PA), and Tobacco Environments and Truckers’ Related Patterns and Practices in the United States and Canada (n = 38).

First Author, Year	Study Objective	Location Country	Recruitment or Sampling Strategy	Inclusion Criteria	Sample size
Angeles, 2014	To examine risk factors and health needs of Canadian truck drivers	Southwestern Ontario, Canada	Companies were identified through the Hamilton Chamber of Commerce and the Ontario Trucking Association	Drivers at companies with at least 10 short haul truck drivers employed	406 truck drivers
Apostolopoulos, 2011	To examine trucking environments and their relationships to dietary behaviors and choices in truckers at high risk of obesity and weight gain	South Central North Carolina, USA	Truck sites were chosen to represent varied geographies and sizes close to the highway and areas where truckers spent time	Truck stops including underserved trucking areas	8 trucking terminals, 7 warehouses, 8 truck stops, 2 highway rest areas
Apostolopoulos, 2012	To examine how the environmental attributes of trucking worksites influence the physical and recreation activity patterns of truckers	North Carolina, USA	Truck sites were chosen to represent sites of varied geographies and sizes that were close to the highway and areas where truckers spent time	Trucking settings located near highways I-85 and I-40	8 truck stops, 8 trucking terminals, 7 warehouses, and 2 highway rest areas
Apostolopoulos, 2013	To explore the connection between truck drivers' work environments and their physical health, access to healthcare services and medical treatment, and participation in health promotion programs	Central North Carolina, USA	316 truckers randomly selected from truck stops and trucking terminals in North Carolina and invited to participate in the Healthy Trucker Survey	Male long-haul truck drivers	316 truck drivers
Apostolopoulos, 2016	To examine the resources and barriers of trucking worksites that can influence health behaviors	North Carolina, USA	Based on geographic and corporate representativeness, proximity to the highway, and worksite size	Trucking settings located near highways I-85 and I-40	8 truck stops, 8 trucking terminals, 7 warehouses, and 2 highway rest areas
Bachmann, 2018	To assess the general and sexual health of long-haul drivers in the U.S.	North Carolina, Tennessee, and Mississippi, USA	Discreet multiple-day site visits for recruitment of eligible participants	Long-haul truckers (a driver, full or part-time, drove a truck across state lines-interstate), English speaking, age 21+	266 long-haul truck drivers
Birdsey, 2015)	To compare selected health behaviors and body mass index (BMI) of US long-haul truck drivers to the US working population by sex	48 contiguous states, USA	Researchers used a weighted sampling process with three stages: first, selecting highway sections based on geographical region and traffic volume; second, selecting truck stops located in the chosen highway sections; third, recruiting long-haul drivers who entered the truck stops	Long-haul drivers were selected if they drove a truck as a main occupation with ≥ 12 months experience, drove a truck with ≥ 3 axles requiring a Commercial Driver’s License, and took ≥ 1 mandatory 10-hour rest period away from home on each delivery run	1,265 long-haul truck drivers
Crizzle, 2020	To examine risk factors associated with and predictive of depressive symptoms	Saskatoon, Saskatchewan; Lloydminster, Edmonton, Red Deer, Calgary, and Alberta, Canada	Drivers entering the truck stop were approached and invited to participate in the study	Long-haul truck drivers who were Canadian and carried a Class 1 or equivalent driver’s license, and spent one or more nights away from home	238 long-haul truck drivers
Crizzle, 2020	To understand where fatigue-related accidents occur and long-haul truck drivers' perceptions about fatigue and truck stop access	Saskatchewan and Alberta, Canada	Truck drivers were approached for interviews by researchers	Long-haul truck drivers who were Canadian and carried a Class 1 or equivalent driver’s license, and spent one or more nights away from home	67 long-haul truck drivers
Gay Anderson, 2008	To explore aspects and prevalence of violence experienced by truck drivers while working	Boston, MA; Dallas, TX; Louisville (and two other sites in Kentucky), KY; Las Vegas, NV; Portland, OR; Chicago, IL; and Des Moines, IA in the USA	Researchers collected surveys from truckers who met the inclusion criteria at truck shows and truck stops across the U.S.	Drivers who spent 1 or more nights away from home, carried a Commercial Driver’s License, were 21 years or older and spoke English	987 long-haul truck drivers
Heaton, 2015	To describe caffeine consumption among truck drivers in the on– and off duty conditions and explore the associations between caffeine use and critical safety events in habitual caffeine-consuming drivers by age in the naturalistic work setting	Alabama (study location was not directly specified), USA	Participants recruited from four trucking companies	Long-haul truck drivers and line haul (e.g., ‘out and back’ routes) truck drivers	97 participants
Hege, 2019	To explore the connection between work characteristics, job stress, sleep outcomes, health behaviors, and physical and mental health outcomes among long-haul truck drivers	Central North Carolina, USA	Signs about the study were distributed around a large truck stop. Researchers used an intercept technique to approach drivers inside the truck stop and screen drivers	Truck stops were chosen based on location to major interstate system (I-40) and amenities for truck drivers	260 long-haul truck drivers
Holmes, 1996	To design and implement a driver nutrition program	Iowa (study location was not directly specified), USA	Researchers mailed surveys (response rate was 21%)	Truck drivers	300 truck drivers
Johnson, 2021	To explore long-haul truck drivers' healthcare experiences and their relationship with health care providers	Toronto, Canada	Researchers recruited truckers from truck stops of interest and using social media postings and snowball sampling	Drivers of long-haul routes with trucks having 3 or more axles. Having driven as a main job for the preceding year and residing in Ontario.	13 long-haul truck drivers
Korelitz, 1993	To assess personal characteristics, health status, and health interests reported by long-haul truck drivers	29 states, USA	A convenience sample, consisting of truck drivers who visited a health booth at one of 65 truck stops participating in the trucker trade show	Male and female long-haul truck drivers. Truckers were away from home overnight each week, and able to read, write, and speak English	2,945 long-haul truck drivers
Layne, 2009	To determine if health conditions and health care access differ between male and female long-haul truck drivers	London, Beaverdam, and Jeffersonville, Ohio, USA	Researchers visited truck sites 5 or more times to recruit 25 males and 25 females. Flyers were used to engage truckers to participate and direct eligible participants to the nearby recreational vehicle and were posted on the fuel islands	All truckers were licensed long-haul truck drivers, away from home overnight each week, and were able to read, write, and speak English	50 long-haul truck drivers
Lemke, 2016	To investigate truckers' difficulties of engaging in favorable health behaviors	Texas, USA	Participants were identified via contact with a key informant who served as a gatekeeper, social media websites (Facebook), and snowball sampling	Currently employed long-haul truck drivers	12 long-haul truck drivers
Lincoln, 2018	To collect data on the built environment of trucking settings and the contribution to the emotional and physical wellbeing of drivers	Minnesota, Ohio, Louisiana, Arkansas, and California, USA	A national list of truck stops located near high-volume traffic highways was used to select stops that had a restaurant, at least 5 paved parking spaces, proximity to facilities, and the ability to combine multiple truck stop visits into single travel events	Truck stops and nearby venues	16 truck stops, including 2 full-service restaurants, 3 fast-food restaurants, and 2 grocery stores
McDonough, 2014	To design and administer a health and wellness survey that describes lifestyle issues affecting health and disease risk factors among truck drivers and perceived by their managers in the truck driving occupation	Hamilton, Ontario, Canada	The transport company occupational health and safety representatives invited focus group participants	Company drivers	16 truck drivers
McGuirt, 2019	To determine the availability, accessibility, and healthfulness of foods and beverages along highway rest areas	North Carolina, USA	Researchers used the North Carolina Department of Transportation website to identify all rest areas in North Carolina and selected locations offering food or beverages for sale	Only toll-free highway rest areas were examined (24 rural, 6 urban)	241 vending machines
Mullins, 2013	To validate the Health Promoting Lifestyle Profile II with Hispanic male truck drivers and assess differences based on occupational driving responsibilities	Southern New Mexico, USA	Data collected at one large truck stop, two smaller truck stops, and a university parking lot. Potential participants were approached and informed about the study	Both long-haul truck drivers (spend most of the year away from home) and short-haul truck drivers (return home in the evening, have a regular schedule, and work 50 or more hours/week)	48 drivers
Olson, 2009	To evaluate the effectiveness of a new health promotion model, the Safety & Health Involvement for Truckers to reduce body weight and increase healthy behaviors	Pacific Northwest, USA	Recruitment by posters and company communications in the regional terminal of each of 4 trucking companies	Truck drivers were prescreened for cardiac risk and had a BMI greater than 26	29 truck drivers
Olson, 2016	To evaluate the effectiveness of the Safety and Health Involvement for Truckers model that includes weight loss competition, behavior and body weight self-monitoring, computer-based training, and motivational interviewing using an RCT design	Oregon (study location was not directly specified), USA	Trucking companies had a driver employment level ranging from 500 to greater than 2000, provided interstate transportation services, and operated at least 2 larger terminals (about 80 + drivers each). Recruitment through personal contacts, referrals, and phone calls.	Employed commercial truck drivers with a BMI ≥ 27	452 drivers
Olson, 2016	To identify clusters of drivers with similar patterns, affecting energy balance (sleep, diet, and exercise) behaviors and test for clusters’ differences in health and psychosocial factors	Western, Midwestern, and Southeastern USA	Drivers were recruited from company terminals using printed advertisements, mailings, announcements at safety meetings, and direct satellite messages	A BMI ≥ 27, an interest in managing or losing weight, and the absence of contraindicating health conditions	452 drivers
Passey, 2014	To explore truck drivers’ views on diet, PA, and health care access to inform the development of a weight loss intervention	USA (Not specified)	Participants were approached at a local truck stop to participate in focus groups.	Required to be an actively employed long-haul truck driver with a current Commercial Driver’s License and ≥ 18 years old	30 long-haul truck drivers
Ronna, 2016	To assess the relationship between the Framingham Cardiovascular Disease Risk Score and the prevalence of U.S. Department of Transportation reportable crashes in commercial motor vehicle drivers, after controlling for potential confounders	Utah, Wisconsin, Kentucky, Texas, Nevada, and Iowa, USA	Convenience sampling used at truck stops and professional truck shows	Commercial truck driver with a current Commercial Driver’s License at the time of study enrollment	797 commercial truck drivers
Shattell, 2010	To identify occupational stressors and the availability of mental health promotion/prevention services	Southeastern region, USA	Truck drivers meeting the inclusion criteria were selected from information collected as part of a larger study	Long-haul truck drivers involved in illicit behaviors	59 long-haul truck drivers
Shattell, 2012	To explore mental health risk factors and preventative services and a possible link between mental health illness and truckers' environments	Greensboro, North Carolina, USA	Researchers asked initial screen questions to every 5th truck driver entering a truck stop	Male truck drivers that were at least 20 years of age	316 truckers
Sieber, 2014	To complete a formative evaluation of long-haul truck drivers’ demographics, working conditions, health risks, and lifestyle related chronic diseases	New Hampshire, New York, Ohio, Michigan, Indiana, Minnesota, Virginia, South Carolina, Tennessee, Florida, Arkansas, Louisiana, Iowa, Nebraska, Kansas, Texas, Oregon, California, and Arizona, USA	Truck drivers were recruited during a 3-day interview period in different 8 h shifts between 7am and 10 pm. Recruiters were instructed to approach all individuals entering when interviewers were available, so each truck driver who entered during a recruitment period had an equal chance of selection	Long-haul truck drivers who drive a truck with ≥ 3 axles as their main job for ≥ 12 months and take at least one mandatory 10-hr rest period away from home during each delivery	1,265 Long-haul truck drivers
Solomon, 2004	To assess health care access	Knoxville, TN; Glade Spring, VA; Girard, OH; Rochelle, IL; Portage, WI; Walcott, IA; Des Moines, IA; Grand Island, NE; Big Springs, NE; Belgrade, MT; Laramie, WY; Commerce City, CO; Oak Grove, MO; Effingham, IL; Carlisle, PA; and Elkton, MD in the USA	Researchers approached truck drivers in truck stops	Long-distance drivers with overnight routes, who resided in the U.S. and drive multiple routes, i.e., “not a dedicated run”	521 truck drivers
Staško, 2007	To explore health care access problems faced by long-distance truck drivers	Michigan, USA	Truck stops were identified and telephoned to determine if the truck stop had an eating area. Letters were mailed to truck stops to describe the study and ask for permission to interview a sample of truckers who met the inclusion criteria. Interviews with truckers were performed while truckers ate their meals	The truck stop needed to have a booth for sitting and eating to ensure a confidential setting. Drivers were required to be long-haul truck drivers	30 long-haul drivers
Thiese, 2015	To assess feasibility of a 12-week weight loss intervention for truck drivers with a weight loss goal of 10% of initial body weight	Utah, USA	Recruitment strategies included hanging fliers at local truck stops, contacting drivers from a previous study, and working with a local truck company to approach eligible drivers	Drivers were current long-haul commercial motor vehicle drivers with a BMI ≥ 30 and age 21 or older	12 long-haul drivers
Turner, 2011	To examine exercise habits and perceived barriers to exercise	Kentucky, USA	A convenience sample of 300 commercial long-distance drivers who operate heavy trucks or tractor-trailers recruited from six truck shows between 2008 and 2009	Working as a commercial truck driver for at least 2 years, 23 years or older, English speaker, a minimum of 2 days overnight on the road per week or 8 days overnight per month, free of infection and other illnesses within the last 2 weeks, did not have an implanted cardiac pacemaker or other implanted device and not pregnant	297 commercial long-distance drivers
Van Hemel, 1998	To assess knowledge and facts about fatigue in truck drivers and identify misconceptions related to the subject	New Mexico, USA	The researcher used the Trucking Research Institute and the American Trucking Associations Foundation that were members of the National Private Truck Council, the Owner Operator Independent Drivers Association, and the Canadian Trucking Association to identify companies interested in participating in the survey and distribute surveys to eligible truck drivers	Long-haul truck drivers who were away from home overnight and often drive long, irregular hours, exposing themselves to a high risk of fatigue	4,833 long-haul truck drivers
Versteeg, 2018	To highlight the level of knowledge truck drivers have on health and the barriers and facilitators to improving their health	Canada (online forums)	Online posts in the Driver Health Forum from the Truckers Report website from 2006 and 2016 were included	Professional truck drivers	1,760 forum discussion posts by truck drivers
Wenger, 2008	To interview truck drivers to gain insights and investigate occupational health and work issues	Massachusetts, USA	Online posts on Craigslist to look for truck drivers and interview them. Received written interview responses	Truck drivers	42 truck drivers
Whitfield Jacobson, 2007	To compare truck drivers' anthropometrics with the recommended guidelines and assess eating/exercise habits, importance of healthful food choices, and attitudes about healthful choices in restaurants	Midwestern Illinois, USA	Researchers used a convenience sample of 100 truck drivers at a Midwestern franchised truck-stop restaurant	Truck drivers, including all races, colors, religions, national origins, ancestry, sex, marital status, or sexual orientation were considered	92 truck drivers
Williams, 2017	To understand the stressors that may contribute to truck driver job turnover	USA	Researchers approached drivers entering truck stops and blogs were identified using online searches	Professional truck drivers	71 truck drivers (61 from interviews, 10 from blogs)

### 3.1 Trucking environments

Four of 38 studies (11%) examined trucking food or PA environments (Table 2) (Apostolopoulos et al., 2012, Apostolopoulos et al., 2011, Lincoln et al., 2018, McGuirt et al., 2019). No studies assessed tobacco environments.

**Table 2:** Trucking Food and Physical Activity (PA) Environment Results in the United States and Canada (n = 4 studies).

Author, year	Food	PA	Measure	Key Results
Apostolopoulos, 2011	**X**		The Healthy Trucking Worksites Audit Instrument (HEATWAI) is a 250-item tool developed from existing measurements used to measure environmental aspects of trucking settings related to food and physical activity behaviors.	**Food:** Among all trucking sites measured, 18.1% were classified as “not at all supportive” of healthy options. Healthy choices available included vegetarian dishes, 100% ‘natural’ juices, and lower priced reduced portions compared to full portions. Fast-food settings available in truck stops scored 22 of 144 possible points (15.3%) for healthy item availability such as salads, vegetable-based soups, and reduced kcal/fat-free condiments. There was an average of 3.1 vending machines at truck stops, 5.2 at trucking terminals, 4.7 at warehouses, and 7.5 at highway rest stops. Vending scores were 225 out of 1,250 possible points (18%). Healthy items in vending machines included nuts, seeds, 100% juices, low-fat milk, and yogurt. Food stores and mini marts at truck stops scored 20 out of 168 possible points (11.9%). Lunch-break rooms and driver lounges that were in trucking terminals, warehouses, and truck stops scored 69 out of 334 possible points (20.7%). Social and informational environments among trucking work settings and in surrounding communities scored 41 out of 209 possible points (19.8%).
Apostolopoulos, 2012		**X**	The HEATWAI is a 250-item tool developed from existing measurements used to measure environmental aspects of trucking settings related to food and physical activity behaviors.	**PA:** Trucking worksites scored 94 of 325 (28.9%) on physical and recreational areas in natural environments (wooded areas and grass spaces), 88 of 330 (26.7%) on physical and recreational areas in built environments, 126 of 469 (26.9%) on resources and facilities that encouraged physical and recreational activities, 0 of 437 on exercise and fitness facilities, 23 of 276 (8.2%) on social environments, 24 of 125 (19.2%) on the promotion of physical and recreation activities (bulletin boards, posters, etc. containing informational messages or opportunities), and 46 of 325 (14%) on physical and recreational amenities and opportunities in nearby community settings.
Lincoln, 2018	**X**	**X**	A checklist was developed to measure availability of convenience store healthy food options and energy products (e.g., energy drinks, shots, or pills), restaurant/fast-food healthy food options, and indoor and outdoor truck stop amenities.	**Food**: Truck stops were found deficient regarding promotions for and availability of healthy items. All truck stops had at least 1 convenience store and either a full-service or fast-food restaurant on site. Of truck stop restaurants, 16 (94%) offered a healthy animal protein and 12 (75%) offered a healthy vegetable. White-meat poultry was the most common healthy item offered in 14 (88%) truck stops, with fish or shellfish less common in comparison. 12 (75%) offered both vegetarian dishes and healthy animal protein dishes and 1 (6%) had no healthy items available. Among convenience stores, 12 (75%) offered a healthy fruit, 7 (44%) offered a healthy snack, 7 (44%) had frozen/canned/dried fruit with no sugar or fat added, 1 (6%) offered healthy vegetable, and none offered a healthy prepared entrée. 1 truck stop stop (6%) offered a healthy vegetable or prepared entrée or both a healthy fruit and vegetable entrée, 3 truck stops or 19.2% did not offer a healthy option including snacks. Lastly, 10 stops or 63% offered a fresh vegetable in either the restaurant or convenience store. **PA**: Only showers (in comparison to other laundry and hygiene amenities) were available in all truck stops; more than 81% lacked a walking path. None offered exercise facilities.
McGuirt, 2019	**X**		The Nutrition Environment Measures Survey–Vending (NEMS–V) measures type, price, and size of foods and beverages and the healthfulness of items offered in vending machines. The scoring criteria used a medal for vending machines based on healthy properties: bronze, at least 30% of food or 55% of beverage items were yellow or green; silver, at least 40% of food or 65% of beverage items were yellow or green; or gold, at least 50% of food or 75% of beverage items were yellow or green. Green foods represented healthy choices, yellow foods were less healthy choices, and red foods were the least healthy choices.	**Food**: Most (n = 12) rest areas had no vending machines that received a healthfulness award. Eleven stops had a gold-award machine, ten had a bronze-award machine, and three had a silver-award machine. Cold beverage machines were more likely than snack machines to receive a bronze, silver, or gold medal (p < 0.001). No differences between awards by location or advertisements for healthy or unhealthy products were found. The average food/beverage items available in vending machines was 24 ± 14.3. The average food item portion size was 1.9 ± 1.1 oz. and 17.2 ± 4.0 oz. for beverage items. The mean price of food items was $1.19 ± $0.39 and $1.63 ±$0.51 for beverages. Most items (61.8%) in cold beverage machines were categorized as red items. The most common “green” beverage was plain bottled water, and the most common “yellow” beverage was Diet Pepsi. Among 19 hot beverage machines, 70.2% were categorized as red due to coffee drinks with both added sugars and creamers. Among five combination machines (e.g., refrigerated and nonrefrigerated beverages and snacks), 55.6% were categorized as red. Among four ice cream machines, 98.0% of items were categorized as red. Among 88 snack machines, 88.6% were categorized as red. Most (99%) snack machines received no award. Most (88%) food items were categorized as red. The most common “green” food was salted peanuts, followed by trail mix, and Goldfish crackers. The 5 most common “red” foods were Skittles, Lays potato chips, Doritos, Snickers, and Peanut M&Ms.

#### 3.1.1 Food environments

Three studies reported data from the measurement of trucking food environments including truck terminals, warehouses, truck stop restaurants, convenience stores, and travel stop vending machines (Apostolopoulos et al., 2011, Lincoln et al., 2018, McGuirt et al., 2019). Lincoln et al. (Lincoln et al., 2018) found most of the sampled restaurant and convenience store settings at truck stops to have fruits or vegetables available for purchase (Lincoln et al., 2018). Overall, however, trucking environments were characterized as ‘unhealthy’ given products with high amounts of saturated fat, added sugars, and sodium were found widely available (Apostolopoulos et al., 2011, Lincoln et al., 2018, McGuirt et al., 2019).

#### 3.1.2 Physical activity environments

Two studies measured PA environments at truck stops (Apostolopoulos et al., 2012, Lincoln et al., 2018). Both found most trucking environments to have showers available (Apostolopoulos et al., 2012, Lincoln et al., 2018) and to lack resources and facilities that encourage PA, including walking paths and exercise centers. Apostolopoulos et al. (Apostolopoulos et al., 2012) also assessed communities near truck stop locations and found most were without PA amenities (Apostolopoulos et al., 2012). See Table 2.

### 3.2 Perceptions of trucking environments

Fifteen of 38 studies (39%) included data about truckers’ perceptions of their food or PA environments (Table 3) (Apostolopoulos et al., 2013, Crizzle et al., 2020, Hege et al., 2019, Holmes and Power, 1996, Layne et al., 2009, Lemke et al., 2016, McDonough et al., 2014, Passey et al., 2014, Staško and Neale, 2007, Johnson et al., 2021, Shattell et al., 2012, Williams et al., 2017, Turner and Reed, 2011, Versteeg et al., 2018, Wenger, 2008, Whitfield Jacobson et al., 2007). No studies explored truckers’ perceptions of tobacco environments.

**Table 3:** Results About Truckers’ Perceptions of Trucking Food and Physical Activity (PA) Environments in the United States and Canada (n = 15).

Author, year	Food	PA	Tobacco	Measures	Key Results
Apostolopoulos, 2013	**X**	**X**		Healthy Trucker Survey (HEATS) that included question on work history, physical and mental health, and healthcare access.	**Food**: Of truckers interested in receiving health information, about half (53.8%) were interested in healthy eating. **PA**: Of truckers interested in receiving health information, about half (49.5%) were interested in exercise guidelines. Fitness facilities were reported unavailable for over 70% of truck stops, 70% of trucking terminals, and 88% of trucking warehouses.
Crizzle, 2020 (7)	**X**	**X**		Interviews using open-ended questions about availability and access to truck/travel stops and driver fatigue.	**Food**: Drivers indicated a lack of rest areas regarding limited breaks for coffee. At times, truckers reported parking in unsafe locations (e.g., near the highway) and walking for food. **PA:** Parking limitations caused needing to utilize unsafe or not well-lit locations for rest stops. Many truck stops were described as not having showers or running water or were described as dirty.
Hege, 2019	**X**			Self-reported daily time spent cooking/eating.	**Food**: 74.2% reported having a few minutes to less than an hour for eating and cooking and 25.8% reported having an hour or more.
Holmes, 1996	**X**	**X**		Situation survey that asked about drivers’ current health and nutrition habits, including healthy food availability on the road along with several nutrition attitude questions.	**Food**: 89% of drivers agreed that they should eat healthier on the road and 83% noted they would order a healthier food choice if available. Only 54% indicated an ability to eat healthfully while on the road. To eat healthier, most drivers indicated needing more time, followed by more healthy foods available, higher income, lower food prices, more nutrition knowledge, knowledge of where to eat, and information about carrying food in the cab. Barriers to maintaining a healthy weight were the abundance of high-fat options and limited healthy food availability. **PA**: Lack of exercise was a noted barrier to weight management.
Johnson, 2021	**X**			Semi-structured interviews included questions about healthcare access and healthcare experiences.	**Food**: Drivers reported difficulty in obtaining healthy foods as a barrier to a healthy lifestyle *“It's what you eat. It's your diet. You gotta realize you're sitting on your ass for 13 hours a day and then you're going to lay in a bunk so what are you burning really?*…*I mean let's be honest. You go sit down in that restaurant you're not going to eat the healthiest food, regardless if you eat a salad or not. It's not healthy.”*
Lemke, 2016	**X**	**X**		Semi-structured interview guide included questions to understand health behaviors, including barriers to consuming nutritious foods and exercising in truck settings.	**Food**: Job stress was perceived to influence food purchasing decisions and prevented engaging in healthy behaviors. Restaurants were perceived to have healthy options on menus; however, were overpowered by unhealthy options, “*Right where the salads are, right above it, are huge slices of pizza* … *When you get a sale on two slices of pizza, you are going to grab that instead of the four-dollar salad that’s below it*.” The abundance of unhealthy food in truck stops was a noted barrier. Factory-installed appliances in truck cabs (e.g., refrigerator/freezer) were a noted facilitator that could enable drivers to carry their own healthy food. **PA**: Drivers perceived making time for exercise as important for health. Drivers recommended resources like workout facilities and walking trail maps to improve PA. One driver suggested “[*Truck stops*] *can have maps with walking trails* … *It wouldn’t cost much money for them to put that on the wall and maybe it would inspire a culture.*”
McDonough, 2014	**X**	**X**		Semi-structured interview questions based on modifiable chronic disease risk factors in truck drivers.	**Food**: Drivers perceived the limited accessibility, availability, and affordability of healthy foods to be related to poor nutrition. Time pressure, parking issues, and overpriced healthy options were also perceived to reduce truckers' diet quality. Truck stop food options were described as lacking in fruit and vegetables with many products that were high in saturated fat, sodium, and kcals. One driver noted, “*Parking is an issue so you can’t get proper food. You go to a truck stop you know*…*and look at the menu. Everything is like ‘drips with grease’*…*.*… *and if they do offer fresh fruit... its way over-priced*.” **PA**: Drivers recognized the importance of PA, but described time constraints, long working hours, and fatigue as barriers. One driver said, “*it’s hard to have a healthy lifestyle when you’re working 16 hours a day*…”
Passey, 2014	**X**	**X**		Focus group discussion guide based on PRECEDE-PROCEED model with questions about access to nutrition information and barriers to eating healthy and PA.	**Food**: Drivers described difficulty storing healthy food in trucks without a refrigerator and noted the vibrations in the cab bruised or damaged fresh produce. One driver noted “*You can find anything you want on the road, some drivers don’t have microwave, and some don’t have coolers. You can go anywhere you want it’s just the storage*.” Parking was a major concern, due to the difficulty in parking in smaller spaces to access healthy options (e.g., near grocery stores). For example, “*a lot of supermarket parking lots are not truck friendly*.” Limited time for food shopping and preparation were also barriers due to long working hours. **PA**: Most drivers described time constraints and a lack of energy or motivation for PA. Gym membership costs and parking spaces near gyms also prevented PA on the road. Truck cabs were described as too constrained for doing exercises (e.g., “*you can’t do them in the truck, there’s not enough room*.”) and parking lots were perceived unsafe or dirty. Poor access to showers and gym facilities in truck stops were also reported barriers. Threat of injury during exercise was also a concern, given long drives to destinations. Irregular sleep schedules also were perceived to unfavorably influence energy for engaging in PA.
Shattell, 2010		**X**		Interviews with open-ended questions about sexual and substance abuse, trauma and coping, mental health, occupational stress, and access to care.	**PA:** Truckers described violence and dangers around truck stops (which may influence PA patterns/practices)
Staško, 2007	**X**	**X**		Interview questionnaire with open-ended questions about health care on the road.	**Food**: Drivers expressed interest in healthier food options and nutrition education. **PA**: Drivers expressed interest in access to exercise rooms.
Turner, 2011		**X**		Obesity Risk Factor Questionnaire including prompts about barriers to exercise, availability of exercise equipment while traveling, and the use of equipment.	**PA**: 75% of drivers rated the exercise environment in a typical work week as “never available/terrible” and 75% also reported exercise equipment was not available on the road. 59% reported they would use an exercise room with weight-lifting equipment and aerobic machines if available at truck stops. When exercise equipment was available, 59.7% reported never using it, 30.7% used it some of the time, and only 9% used it always or most of the time. 72.7% said home exercise was easier than when traveling. Barriers to PA were lack of time (66.7%), lack of exercise facilities (45.3%), safety concerns (7.3%), physical health limitations (6.3%), cost (4.3%), and crowded exercise facilities (3.7%). 71% reported not having enough time when exercise facilities were available. Drivers with obesity who perceived exercise equipment as not available reported no availability of exercise facilities and rated the exercise environment as terrible or bad (p = 0.01) and reported difficulty exercising due to personal health (p = 0.03) in comparison to drivers without obesity. Drivers who perceived that exercise equipment was available were 2.58 times more likely to exercise 30 minutes a day for 5 days while traveling (p = 0.001). Drivers who exercised 5 days or more were 0.30 times less likely to report not having time to exercise (p = 0.001). Drivers who exercised at least 1 day in the past week were 0.49 times less likely to have been traveling for work for 5 days or more in the past week (p = 0.01).
Versteeg, 2018	**X**	**X**		A content analysis was used to assess posts to the Driver Health Forum from the Truckers Report website.	**Food**: 56% of truckers posted about health topics and 26% posted about regulations affecting health. Drivers lacked knowledge of healthy eating as evidenced by the types of questions posted on the forum and responses (i.e., about using fad diets, weight loss pills, and surgery to lose weight). Posts were often related to losing weight, healthy eating strategies, and cooking on the road. Many posted about barriers to healthy eating, including unhealthy food in truck stops, “*For starters stay away from the truck stop food. That’s where most gain weight from all the greasy garbage truck stops call food*.” The need for a lot of caffeine to stay awake on long drives and limited time on the road to cook were noted. There were a high number of posts about food (indicating topic importance). **PA**: Many forum posts were about exercising in the trucks, gym memberships, and suggestions from other drivers regarding running or walking trails near truck parking lots. Some were interested in learning about PA options, “*I have been jumping around this forum looking for practical ideas. Unfortunately, most threads seem to lose steam rather quickly*.” Truckers were also looking for quick and effective PA options and demonstrated a lack of knowledge about exercise.
Wenger, 2008	**X**	**X**		Truckers responded to three questions through interviews and written responses: 1) What made you choose your profession?; 2) What are your concerns (if any) as a driver?; and 3) What do you like most and least about your job?	**Food**: A lack of healthy food options relative to unhealthy options was expressed, “*A major issue I had was that none of the truck stops had any good, nutritious food. You would eat fatty, fried foods and have heartburn and have to sit in your truck for 11 hours straight*. *Nothing was nutritious, only things like steak and mashed potatoes and gravy*.” **PA:** Drivers described the sedentary nature of the job in relation to health outcomes, “*Health is a major factor sitting on your butt for 11 to 18 hours a day. Most drivers are so extremely out of shape that they find them on the side of the road with a heart attack. No joke either*.” Long drive hours were also described to contribute to fatigue and reduced motivation for PA, *“Drivers are usually expected to sit and drive for 11 hours a day. This doesn’t leave much time for exercise, nor do places where drivers stop help with exercising either. And after driving for 11 hours, you just want to eat a meal, take a shower, and go to sleep or watch TV. Only a few drivers make an effort to carry exercise equipment in the truck.*”
Whitfield Jacobson, 2007	**X**			Self-reported days per week engaged in 30 minutes sustained duration of exercise; self-reported average fruit/vegetable consumption per day. Food Choices Index (FCI) was used to measure the importance of healthy food choices. The Nutrition Attitude Survey (NAS) was used to measure attitudes about truck stops restaurant provision of healthful foods.	**Food**: Drivers who valued healthful options were more likely to choose nutritious options in restaurants if available (*p* < 0.001). Drivers perceived taste as more important than nutrition when making dietary choices. Regarding the importance of healthy choices, truckers’ mean FCI score (higher number, higher importance) was 56.94 ± 7.66 (range 15–75). NAS scores about healthy restaurant options were on average 44.84 ± 5.68 (range 12–60). No differences were found between drivers with normal weight or overweight/obesity regarding reported importance of healthful food choices (p = 0.193). Drivers noted policy and environmental opportunities for improving dietary patterns: promoting healthful menu items in truck stop restaurants; achieving richer flavor without added fat or salt; using nutritious ingredients and lower kcal, fat, and cholesterol options; increasing low kcal menu items; centering nutrition concerns in menu development; and nutrition education.
Williams, 2017	**X**	**X**		Short informal interviews included open-ended questions, e.g., “Can you tell me about your experience as a truck driver?”. Web blogs used by truckers were used to identify challenges of the trucking profession.	**Food:** Drivers reported less access to healthy food as a barrier to choosing healthy choices when being on the road for a long time. Truck drivers reported that driving tends to encourage picking up fast-food and drinking lots of caffeine. **PA:** Drivers reported limited time to exercise when being on the road for a long time. *“Some do choose to exercise frequently, but the demands placed on an over-the-road trucker tend to limit these activities.”*

#### 3.2.1 Food environment perceptions

Thirteen studies had results about truckers’ perceptions of the trucking food environment (Apostolopoulos et al., 2013, Crizzle et al., 2020, Hege et al., 2019, Holmes and Power, 1996, Lemke et al., 2016, McDonough et al., 2014, Passey et al., 2014, Staško and Neale, 2007, Johnson et al., 2021, Williams et al., 2017, Versteeg et al., 2018, Wenger, 2008, Whitfield Jacobson et al., 2007). Environmental barriers to healthy eating were, in majority, described regarding the wide accessibility of less healthy food and beverage products with few healthy options in comparison (Holmes and Power, 1996, Lemke et al., 2016, McDonough et al., 2014, Staško and Neale, 2007, Johnson et al., 2021, Williams et al., 2017, Versteeg et al., 2018, Wenger, 2008, Whitfield Jacobson et al., 2007). Job-related stress or time barriers (Apostolopoulos et al., 2013, Hege et al., 2019, Holmes and Power, 1996, Staško and Neale, 2007, Versteeg et al., 2018, Whitfield Jacobson et al., 2007) and a lack of nutrition knowledge (Holmes and Power, 1996, Lemke et al., 2016, McDonough et al., 2014, Passey et al., 2014, Versteeg et al., 2018) were also described as barriers to meeting dietary guidance recommendations in the food environment. Other barriers to healthy eating included cost (Holmes and Power, 1996, Lemke et al., 2016, McDonough et al., 2014), the ability to carry or prepare food in truck cabs (Holmes and Power, 1996, Lemke et al., 2016, Passey et al., 2014), and limited parking space or availability (Crizzle et al., 2020, McDonough et al., 2014, Passey et al., 2014). In one study conducted in Canada, a dearth of environmental options to access food/beverages while on the road was described (Crizzle et al., 2020). See Table 3.

#### 3.2.2 Physical activity environment perceptions

Twelve studies assessed truckers’ perceptions of PA environments (Apostolopoulos et al., 2013, Crizzle et al., 2020, Holmes and Power, 1996, Layne et al., 2009, Lemke et al., 2016, McDonough et al., 2014, Passey et al., 2014, Staško and Neale, 2007, Shattell et al., 2012, Turner and Reed, 2011, Versteeg et al., 2018, Wenger, 2008). Although truckers believed a lack of exercise contributed to weight gain and poor health (Holmes and Power, 1996, Whitfield Jacobson et al., 2007) and recognized the importance of PA (McDonough et al., 2014, Versteeg et al., 2018), there were many reported barriers to PA including lack of time, long and inflexible work hours, and fatigue (McDonough et al., 2014, Passey et al., 2014, Turner and Reed, 2011, Wenger, 2008). Poor or no access to PA facilities and equipment was another primary barrier (Apostolopoulos et al., 2013, Passey et al., 2014, Turner and Reed, 2011, Wenger, 2008). In one study, limited parking resulted in needing to stop in unsafe locations and a lack of showering facilities at available rest stops was described (Crizzle et al., 2020), which may influence the likelihood of PA. Finally, some truckers expressed interest in PA and explained they would use exercise equipment, gym memberships, and walking paths if available in the environment (Lemke et al., 2016, Staško and Neale, 2007, Turner and Reed, 2011, Versteeg et al., 2018).

#### 3.2.3 Food, PA, and tobacco patterns or practices

Twenty-seven of 38 studies (71%) had data about truckers’ food, PA, and/or tobacco patterns or practices (Table 4).

**Table 4:** Results About Truckers Food, Physical Activity (PA), and Tobacco Patterns or Practices in the United States and Canada (n = 21 studies).

Author, year	Food	PA	Tobacco	Measures	Key Results
Angeles, 2014	**X**	**X**	**X**	2008 Canadian Community Health Survey assessed general health, sleep and tobacco use, diet and salt intake using the Healthy Eating Index, and PA through the Metabolic Equivalent Task (low, moderate, or high).	**Food**: 48% of truckers were characterized as having a poor diet. 82% reported very high salt intake (above 2300 mg/day) and over 96% consumed above 1,500 mg/day. 42% reported high alcohol intake. **PA**: 31.1% were physically inactive. **Tobacco**: 31.5% reported cigarette smoking.
Apostolopoulos, 2013		**X**		Healthy Trucker Survey (HEATS), based on Long-Haul Trucker Interview Guide, the Health Appraisal Survey, and the Health Survey of the NSW Transport Industry, included questions on work history, workplace conditions, physical health, wellness, mental health, healthcare access and medical treatment history.	**PA**: 70% of drivers reported not participating in some form of regular exercise.
Bachmann, 2018		**X**	**X**	Self-reported health including PA and smoking status.	**PA**: Differences between company and independent drivers were not statistically significant. 62.8% of company and 50% of independent drivers reported exercising in the past 30 days (p = 0.10). 64.5% of all truckers reported feeling out of shape. **Tobacco**: Differences between company and independent drivers were not significant. Most company (68.3%) and independent drivers (64.6%) reported not smoking (p = 0.61). 47.1% of company and 56.3% of independent drivers reported trying to quit smoking within the last year (p = 0.58).
Birdsey, 2015		**X**	**X**	National Survey of U.S. Long-Haul Truck Driver Health and Injury, including questions about cigarette smoking status, age at which smoking began, cigarettes smoked per day, pack-years of smoking, and total PA including work and leisure time.	**PA**: 73.8% of men (95% CI 68.8–78.9) and 80.5% of women (95% CI 68.0–93.0) reported less than 5 days in the past 7 with 30 min of PA. 28.4% of men (95% CI 24.7–32.2) and 25.2% of women (95% CI 12.0–38.3) reported no exercise. **Tobacco**: The prevalence of current smoking was 46.2% (95% CI 40.3–52.2) among men and 54.9% (95% CI 42.8–67.1) among women drivers. 65.3% of men and 68.3% of women reported smoking at some point in their lives. 19.1% of men (95% CI 15.6–22.6) and 13.4% of women (95% CI 3.4–23.4) were former smokers. Average age at first time smoking was 16.4 years among men (95% CI 16.0–16.7) and 15.4 years among women (95% CI 14.1–16.8). Men reported smoking an average of 18.5 cigarettes/day (95% CI 16.5–20.6) and women 19.2 cigarettes/day (95% CI 15.8–22.6). Mean pack of cigarette/years in men 27.3 (95% CI 24.1–30.5) and women 23.1 (95% CI 18.3–28.0).
Crizzle, 2020	**X**	**X**	**X**	Self-administered survey about health characteristics and depressive symptoms	**Food:** Drivers with depressive symptoms (n = 47) consumed alcohol 0.63 ± 1.21 days/ month (range 0–4 days) p = 0.828. 79% consumed 0 drinks/ day, 15% consumed 1–2, 6% consumed 3–4. 66% consumed a healthy diet and 44% consumed an unhealthy diet. The 60 drivers without depressive symptoms consumed alcohol 0.77 ± 1.99 days / month (range 0–10 days) p = 0.828. 69% consumed 0 drinks / day, 22% consumed 1–2, 7% consumed 3–4, 2% consumed 5–6. 70% consumed a healthy diet and 30% consumed an unhealthy diet. **PA:** Of the 47 drivers with depressive symptoms, PA was reported 3.0 ± 2.3 days/week. Of the 60 drivers without depressive symptoms, PA was reported 2.8 ± 2. days/week (p = 0.638). **Tobacco:** Of the 47 drivers with depressive symptoms, 46% smoked every day, 10% some days, 44% not at all. Of the 60 drivers without depressive symptoms, 43% smoked every day, 7% some days, 50% did not at all (p = 0.774).
Gay Anderson, 2008	**X**			Work related safety and violent victimization survey	**Food:** 63.04% of drivers drank alcohol (beer, wine, liquor) within the past 12 months. 38.79% drink alcohol less than once/month, 36.82% 1–2 times/month, 11.13% several times/month, 11.29% 1–2 days/week, 1.29% daily.
Heaton, 2015	**X**			Self-reported log of caffeinated beverage consumption (ounces/day).	**Food**: Drivers reported drinking an average of 16.4 oz of caffeine drinks when on duty and 15.1 oz when off duty.
Hege, 2019	**X**	**X**	**X**	Self-reported health behaviors regarding the number of alcohol drinks on non-workdays, caffeine intake, smoking status, and daily exercise habits.	**Food**: 51.4% reported not consuming alcohol on non-workdays and 48.6% reported having one or more alcoholic drinks on non-workdays. **PA**: 39.4% of truckers were sedentary and 60.6% were moderately active. **Tobacco**: 48.6% reported smoking.
Holmes, 1996	**X**			Situation Survey that asked about drivers’ current health and nutrition habits, including the number of daily servings of meats, proteins, fruits, vegetables, cereals, breads, and milk products consumed. Also included questions on how often they ate out on the road, what meals were consumed on the road, what determined their meal choice, and their favorite meals and snacks.	**Food**: 55% of drivers ate dinner while on the road Mondays-Fridays (lowest frequencies of all meals on Saturdays/Sundays). 48% of drivers stopped for breakfast on Mondays-Fridays. 44% of drivers ate lunch on the road during weekdays. Snacks were also high during weekdays (range 45–60% of drivers). Drivers reported their favorite meals on the road were steak (29%), burgers (25%), chicken (17%), and a buffet (13%). Drivers most frequently reported snacks were chips, then fruit, followed closely by candy, donuts, and cookies. Less frequently reported snacks in comparison were popcorn, crackers, pop, and ice cream. Most drivers were found to meet daily protein and dairy requirements and only a minority met servings for fruits, vegetables, and cereals and breads.
Korelitz, 1993	**X**	**X**	**X**	Self-administered, close-ended questionnaire that measured participants’ personal characteristics, health status, and health interests.	**Food**: More than 80% reported eating only one or two meals per day and 36% reported consuming three or more snacks each day. 59.2% were current drinkers. **PA**: 49.6% reported never engaging in PA, 39.5% reported sometimes engaging in PA, and 8% reported regular PA. **Tobacco**: 37.7% never smoked, 6.1% were ex-smokers, 17.3% smoked less than 1 pack/day, and 36.7% smoked more than 1pack/day.
Layne, 2009		**X**	**X**	Four-section questionnaire asking about self-perceived health status, health care access, work experience, and health care needs.	**PA**: No exercise was reported among 15 male (60%) and 14 female truckers (58%). Exercising for 1 or 2 days per week was reported among 7 males (28%) and 6 females (25%). Exercising 3 + days per week was reported among 3 male (12%) and 4 female drivers (17%). **Tobacco**: Current smoking was reported among 40% of male and 60% of female drivers. 24% of male and 4% of female drivers were described as formal smokers. 9 or 36% of male and female drivers reported never smoking.
Mullins, 2013	**X**	**X**		The Health-Promoting Lifestyle Profile II (HPLP II) survey, based on Pender’s Health Promotion model, was offered in both English and Spanish and included questions about nutrition, exercise, and health. For all items, a 4-point scale is used (1 = never; 4 = routinely).	**Food:** Scaled responses for eating 6–11 servings of bread, cereal, rice, and pasta/day was 3.28 ± 0.72 (Spanish version) and 1.77 ± 0.69 (English version) (P = 0.02). The study concluded the HPLP II nutrition subscale may not adequately assess behavior among the study population. **PA:** Scaled responses for exercising vigorously for 20 or more minutes at least three times per week (brisk walking, bicycling, aerobic dancing, using a stair climber) were on average 2.64 ± 1.00 (Spanish version) and 2.00 ± 0.91 (English version) (P = 0.04). Responses for taking part in light-to-moderate PA (sustained walking for 30–40 min 5 or more times/week) were on average 3.14 ± 0.89 (Spanish version) and 2.00 ± 1.00 (English version) (P = 0.012). Responses for doing stretching exercises at least 3 times a week were on average 2.57 ± 1.01 (Spanish version) and 1.81 ± 0.87 (English version) (P = 0.02 less than 0.05). The study concluded the HPLP II physical activity subscale may not adequately assess behavior among the study population.
Olson, 2009	**X**	**X**		Dietary behaviors measured using validated measures of daily fruit and vegetable consumption and using “Gear Up for Health” to assess dietary fat and sugar consumption. Exercise behaviors measured using the 7-day PA Recall interview, which was used to convert to active kcals/kg/week of moderate to vigorous PA. Other measures were used to assess fitness: strength (maximum pushups and timed curl-ups; grip strength measured with a hand dynamometer); flexibility (sit-and-reach test); and the 6-minute walk test.	**Food:** 75% of drivers reported consuming a mean of 36.6% kcals from fat over the past month. Mean sugary snack consumption was reportedly 2.55 times/week, mean sugary drink consumption was reportedly 4.22 times/week, and mean fast-food consumption was reportedly 2.32 times/week. 69% reported consuming an average of 3.05 fruit and vegetables servings per day and 76% consumed an average of 2.31 meals from home per week. Among most participants (75%), the frequency of sugary drinks was “5 or 6 times a week, the frequency of sugary snacks was “1 or 2 times a week”, and frequency of fast-food was reportedly “1 or 2 times a week.” **PA:** The mean reported active kcals/kg/week among all drivers was 23.32. Mean active kcal/ kg/week for drivers who reported a *typical* week before interviews (n = 7) was 13.14. Around a quarter of drivers (27%) reported engaging in 40 min of moderate exercise most days each week. On average, the 6-minute walking test results were 525.05 m, the push-up results were 3.36, the valid curl ups were 6.41, and the flexibility results were 12.59 in..
Olson, 2016	**X**	**X**	**X**	Self-reported daily fruit and vegetable intake, percent of calories from fat, and frequency of sugary food, sugary drink, and fast-food consumption. The healthy PA scale was used to measure days/week with 30 min of PA. Demographic information on health behaviors was collected, including smoking.	**Food**: Average daily fruit and vegetable servings/day was 2.9 (SE 0.8) (control group) and 2.63 (SE 0.8) (intervention group). Daily %kcal from fat was reportedly 33.26 (SE = 0.35) (control group) and 33.63 (SE = 0.32) (intervention group). Average sugary snacks/week was 4.15 (SE 0.04) (control group) and 4.14 (SE = 0.04) (intervention group). Average sugary drinks/week was 4.58 (SE = 0.05) (control group) and 4.68 (SE 0.05) (intervention group). Average fast-food consumption/week was 4.01 (SE = 0.04) (control group) and 3.84 (SE = 0.03) (intervention group). **PA**: Average days/week with 30 min of PA was 1.39 (SE = 0.11) (control group) and 1.19 (SE = 0.10) (intervention group). **Tobacco**: 30% of drivers reported smoking in the past month.
Olson, 2016	**X**	**X**	**X**	National Cancer Institute screeners for daily fruit and vegetable servings, dietary fat, and daily sugary snacks and drinks consumed. The Healthy PA Scale measured days/week with moderate to vigorous PA and strength training.	**Food**: Mean fruit and vegetable consumption was reported as 2.6 ± 2.3 servings/day. The proportion of drivers meeting the “5-A-Day” recommendation was 0.09. **PA**: Average days/week with 30 min of moderate to vigorous exercise was 2.7 ± 2.9. Average days/week engaged in strength training was 0.7 ± 1.4. Proportions of the sample meeting recommendations for PA and strength training were 0.24 and 0.20, respectively. **Tobacco**: 30.2% of drivers reported having smoked in the past month.
Ronna, 2016	**X**	**X**	**X**	A self-reported questionnaire to assess smoking, drinking, and PA behaviors. Metabolic Equivalent for Tasks (MET) levels were derived from the amount of time and frequency drivers spent in specific activities (e.g., running, walking).	**Food**: 58.9% of drivers reported drinking alcohol at least once in the past year. **PA**: Drivers reported weekly PA averaging 281 minutes; more than half (57%) reported participating in regular exercise. 102 drivers (12.8%) reported no PA on a weekly basis. Mean MET levels were 12.97 ± 23.6 mL/kg/min. Average time sitting was reported as 4.3 hours/day outside of work. **Tobacco**: 49.6% of drivers reported smoking.
Sieber, 2014	**X**	**X**	**X**	A self-reported questionnaire included questions on work environment, work history, driving practices, and risk factors affecting drivers.	**Food**: 38.9% of drivers reported not drinking alcohol. **PA**: 27.1% reported no moderate or vigorous PA for 30 min in the past seven days. **Tobacco**: 51% reported current cigarette smoking.
Shattell, 2010	**X**			Interviews with open-ended questions about sexual and substance abuse, trauma and coping, mental health, occupational stress, and access to care.	**Food:** 23.7% of drivers never consumed alcohol, 32.2% occasional, 13.6% had alcohol daily (1–12 beers/day); 23.7% had alcohol weekly (mostly weekends) (6–12 packs/week). An additional 5% reported alcohol use, but did not respond to amount.
Shattell, 2012	**X**			The Healthy Trucker Survey that included questions about health, substance use, and health care access on the road.	**Food:** 33.5% of drivers reported the frequency of alcohol use as once a month or less, 20.6% 2–4 times/month, 5.7% 2–3 times/week, 2.2% 4 or more times a week. 11.7% reported 1–2 drinks/day, 5.1% reported 3–4 drinks/day, 1.9% reported 5–6 drinks/day, 0.9% reported 7–9 drinks/day, 2.8% reported 10 or more drinks/day. For drivers who had 6 + drinks at one sitting, 24.7% indicated once a month or less, 7.6% noted 2–4 times/month, 3.2% noted 2–3 times/week, and 0.3% noted 4 or more times a week. 0.9% reported no or <1 drinks first thing in the morning, 0.3% reported 2–4 times/month, and 0.3 reported 2–3 times/week.
Solomon, 2004	**X**	**X**	**X**	Self-administered survey included questions on access to health care, access to health-related educational material, health problems, and recent health complaints.	**Food:** 335 (65%) truckers reported eating healthy on the road. **PA:** 302 (58%) truckers reported exercising on the road. **Tobacco:** 161 (31%) of truckers reported quitting smoking.
Staško, 2007	**X**		**X**	Interview question that included questions about risk factors (smoking and coffee frequency consumption).	**Food**: About 27% of drivers reported consuming over 5 cups of coffee/day and about 47% reported consuming up to 5 cups/day. Around 57% of drivers reported consuming no alcohol. About 20% of drivers reported consuming 6 beers or more/week. **Tobacco**: Around 23% of drivers reported never smoking, 43% reportedly quit smoking, and 33% reported current smoking. Of smokers, 30% reported smoking up to 1.5 packs/day, 20% reported 1.5 to 2 packs/day, and 40% reported 2 packs/day or more.
Thiese, 2015	**X**	**X**		Automated Self-Administered 24- hour Recall by the National Cancer Institute. Minute/week of PA was self-reported.	**Food**: Median total daily kcals consumed by drivers was 3036.5, (IQR = 1792.0), total fat was 133.9 g (IQR = 80.0), saturated fat was 45.7 g (IQR = 31.2), total carbohydrate was 313.4 g (IQR = 140.9) and dietary cholesterol was 511.1 mg (IQR = 342.8). **PA**: Baseline reported PA was 0.0 min/week (SD = 120).
Turner, 2011		**X**		Obesity Risk Factor Questionnaire including 13 questions on exercise habits.	**PA**: 20% reported not engaging in any type of exercise during the past 7 days. More than half reported aerobic exercise 3 days or less during the past week. More than 25% of drivers engaged in no aerobic exercise at all during the past week. The majority (56.7%) reported no stretching or (58%) strengthening exercises in the past week. Nearly half (48.7%) reported not engaging in 30 minutes of continuous exercise on any of the previous weeks. Drivers with a BMI of less than 30 reported more days of stretching exercises on average than drivers with a BMI of 30 or more (p = 0.01).
Van Hemel, 1998	**X**	**X**	**X**	Survey questions about safety, work scheduling, substance use, and overcoming fatigue on the road. Survey included 2 open-ended questions: 1) “What do you do to fight fatigue on the road?; and 2) What works best for you?”	**Food**: To fight fatigue on the road 27.9% of drivers reported eating snacks, meals, or candy, drinking beverages or water, and chewing gum. 21.2% reported consuming caffeine containing foods/drinks (e.g., coffee, tea, Coke, Pepsi, Mountain Dew, Jolt or chocolate) and 5.5% reported eating healthy foods and taking vitamins. **PA**: 34% reported exercising (extent ranged from stretching in the truck, to getting out and kicking tires, to running) and 5.5% reported maintaining fitness (e.g., watch weight, get enough exercise). **Tobacco**: Less than 1% of drivers reported smoking or using other tobacco products (e.g., chewing tobacco, snuff, dip) to fight fatigue.
Versteeg, 2018	**X**		**X**	Driver Health Forum posts from the Trucker Report Website were coded and analyzed.	**Food**: There were 289 (16%) food-related posts, which was the highest number of posts by category. Typical foods consumed were categorized as energy dense and ultra-processed foods. **PA**: There were 85 (4.8%) exercise-related posts. Drivers wanted more opportunities to exercise in trucks and suggested building walking or running trails around truck stops. Offering gym memberships was also a recommendation. **Tobacco**: There were 52 (3%) posts that mentioned smoking or e-cigarette use (no further details). This category received the highest number of replies/posts.
Wenger, 2008		**X**	**X**	Truckers responded to three questions through interviews and written responses: 1) What made you choose your profession?; 2) What are your concerns (if any) as a driver?; and 3) What do you like most and least about your job?	**PA + Tobacco**: Drivers described a lack of PA and smoking more than usual, “*In the 15 plus years I was over the road, I have to say it was good for me in many ways, but I neglected myself in others. I gained weight, got little exercise, and smoked more than I ever did*.”
Whitfield Jacobson, 2007	**X**	**X**		Self-reported average fruit and vegetable consumption/day and days per week engaged in 30 min sustained exercise.	**Food**: Average servings of fruits/vegetables was 1.72 per day (median 1.5/day). About 12% of drivers reported consuming no fruit or vegetable servings/day, 40.2% reportedly consumed 1 serving/day, 22.8% reportedly consumed 2 servings/day, 18.5% reportedly consumed 3 servings/day, and 6.5% reportedly consumed more than 3 servings/day. **PA**: Mean days/week exercised for 30 min sustained duration was reported 2.19 (median 2). 36% reported no exercise, 8.7% reported exercising 1 day/week, 22.8% reported exercising 2 days/week, 8.7% reported exercising 3 days/week, and 24% reported exercising 4 days/week.

#### 3.2.4 Food patterns/practices

Twenty-one studies had data about truckers’ dietary patterns/practices (Angeles et al., 2014, Versteeg et al., 2018, Van Hemel and Rogers, 1998, Heaton and Griffin, 2015, Hege et al., 2019, Holmes and Power, 1996, Korelitz et al., 1993, Mullins et al., 2013, Olson et al., 2009, Olson et al., 2016, Olson et al., 2016, Ronna et al., 2016, Sieber et al., 2014, Staško and Neale, 2007, Thiese et al., 2015, Whitfield Jacobson et al., 2007, Gay Anderson and Riley, 2008, Crizzle et al., 2020, Shattell et al., 2012, Shattell et al., 2012, Solomon et al., 2004). Truckers’ diets were overall characterized as poor (Angeles et al., 2014, Versteeg et al., 2018) – aside from truckers surveyed in two studies (Crizzle et al., 2020, Solomon et al., 2004) – with high amounts of both sodium (Angeles et al., 2014) and %kcal from fat (Olson et al., 2009, Olson et al., 2016, Thiese et al., 2015) in relation to dietary guidance recommendations (Health Canada, 2019, U.S. Department of Agriculture, 2020). Few drivers met recommendations for daily fruit and vegetable consumption (Holmes and Power, 1996, Olson et al., 2009, Olson et al., 2016, Olson et al., 2016, Whitfield Jacobson et al., 2007, U.S. Department of Agriculture, 2020). Truckers reported consuming around 1–2 meals per day in one study (Korelitz et al., 1993), and another found dinner was the most common meal consumed during weekdays on the road (breakfast was less common) (Holmes and Power, 1996). Snacking was frequently reported among drivers (Holmes and Power, 1996, Korelitz et al., 1993).

Truckers reported frequent consumption of meats, salty snacks, fruit, and sweets (Holmes and Power, 1996). Truckers reported consuming sugary snacks, sugar-sweetened beverages, and fast-food at least 2 times per week on average, with sugary drinks reported most frequently (Olson et al., 2009). In another study truckers reported consuming these products about 4 times/week (Olson et al., 2016). Caffeinated beverage intake, including coffee, was frequent (Heaton and Griffin, 2015, Staško and Neale, 2007). For example, the majority of truckers interviewed by Staško et al (2007) (Staško and Neale, 2007) reported drinking up to or more than 5 cups of coffee each day. Caffeinated beverages and snacking were reported to help fight fatigue (Van Hemel and Rogers, 1998).

Eleven studies assessed alcohol intake (Angeles et al., 2014, Hege et al., 2019, Korelitz et al., 1993, Wenger, 2008, Gay Anderson and Riley, 2008, Crizzle et al., 2020, Shattell et al., 2012, Shattell et al., 2012, Ronna et al., 2016, Sieber et al., 2014, Staško and Neale, 2007). The majority of truckers among six studies did not report high alcohol use in general (Angeles et al., 2014, Hege et al., 2019, Staško and Neale, 2007, Crizzle et al., 2020, Shattell et al., 2012, Shattell et al., 2012). In two studies, over half reported drinking alcohol within the past year (Ronna et al., 2016, Gay Anderson and Riley, 2008). A minority reported consuming alcohol weekly (Staško and Neale, 2007, Shattell et al., 2012, Shattell et al., 2012) or daily (Gay Anderson and Riley, 2008). Other studies found most truckers reported being current drinkers (Korelitz et al., 1993, Sieber et al., 2014).

#### 3.2.5 Physical activity patterns/practices

Twenty studies assessed truckers’ PA patterns or practices (Apostolopoulos et al., 2013, Angeles et al., 2014, Hege et al., 2019, Korelitz et al., 1993, Layne et al., 2009, Ronna et al., 2016, Sieber et al., 2014, Thiese et al., 2015, Turner and Reed, 2011, Wenger, 2008, Whitfield Jacobson et al., 2007, Crizzle et al., 2020, Solomon et al., 2004, Bachmann et al., 2018, Birdsey et al., 2015, Van Hemel and Rogers, 1998, Mullins et al., 2013, Olson et al., 2009, Olson et al., 2016, Olson et al., 2016). Nineteen assessed whether truckers engaged in aerobic activity (Apostolopoulos et al., 2013, Angeles et al., 2014, Hege et al., 2019, Korelitz et al., 1993, Layne et al., 2009, Ronna et al., 2016, Sieber et al., 2014, Thiese et al., 2015, Turner and Reed, 2011, Whitfield Jacobson et al., 2007, Crizzle et al., 2020, Solomon et al., 2004, Bachmann et al., 2018, Birdsey et al., 2015, Van Hemel and Rogers, 1998, Mullins et al., 2013, Olson et al., 2009, Olson et al., 2016, Olson et al., 2016). Of these, 13 found that a majority were inactive or insufficiently active (Apostolopoulos et al., 2013, Birdsey et al., 2015, Van Hemel and Rogers, 1998, Korelitz et al., 1993, Layne et al., 2009, Thiese et al., 2015, Turner and Reed, 2011, Whitfield Jacobson et al., 2007, Mullins et al., 2013, Olson et al., 2009, Olson et al., 2016, Olson et al., 2016), while the remaining six found that a majority of truckers were active (but did not differentiate between insufficient or sufficient levels of PA) (Angeles et al., 2014, Bachmann et al., 2018, Ronna et al., 2016, Sieber et al., 2014, Crizzle et al., 2020, Solomon et al., 2004). Two of the studies assessed whether truckers engaged in strength training, and both found that most truckers to not meet strength training recommendations (Olson et al., 2016, Turner and Reed, 2011). Finally, in one qualitative assessment, truckers mentioned getting little exercise (Wenger, 2008).

#### 3.2.6 Tobacco patterns/practices

Sixteen studies reported tobacco use among truckers (Angeles et al., 2014, Bachmann et al., 2018, Birdsey et al., 2015, Crizzle et al., 2020, Hege et al., 2019, Korelitz et al., 1993, Layne et al., 2009, Olson et al., 2016, Olson et al., 2016, Ronna et al., 2016, Sieber et al., 2014, Solomon et al., 2004, Staško and Neale, 2007, Van Hemel and Rogers, 1998, Versteeg et al., 2018, Wenger, 2008). Nine of these only assessed self-reported current smoking (Angeles et al., 2014, Hege et al., 2019, Korelitz et al., 1993, Olson et al., 2016, Olson et al., 2016, Crizzle et al., 2020, Ronna et al., 2016, Sieber et al., 2014, Staško and Neale, 2007), and estimates of current smoking ranged from 30% (Olson et al., 2016, Olson et al., 2016) to 50% (Korelitz et al., 1993, Ronna et al., 2016, Sieber et al., 2014) or more of truckers. Two studies compared current smoking by gender, and both found that female truckers had higher rates of current smoking relative to their male counterparts (46% among men vs. 55% among women in one national U.S. study (Birdsey et al., 2015); 40% among men and 60% among women in a pilot study that collected data from truckers at three rural Ohio truck stops (Layne et al., 2009). Another study found no significant difference in smoking between drivers with depressive symptoms (46% smoked every day) vs. those without (43% smoked every day) (Crizzle et al., 2020).

One study compared smoking rates between independent and company drivers and found no significant differences between groups (though both had high rates of smoking: 47% of company and 56% of independent truckers reported current smoking) (Bachmann et al., 2018). One study assessing sleep behaviors and fatigue among truckers reported that less than 1% of truckers report smoking or using other tobacco products to stay awake on the road (Van Hemel and Rogers, 1998), and another found that roughly a third of truckers in their sample (31%) reported quitting smoking (Solomon et al., 2004). A final study assessing trucker health knowledge using an online forum found that while online posts related to smoking comprised only 3% of posts, the subject of smoking and e-cigarette use resulted in the highest number of reply posts, indicating that truckers were engaged with these topics (Versteeg et al., 2018). Some reported smoking ‘more than ever’ (Wenger, 2008).

### 3.3 Main findings and importance

Available data about trucking food, PA, tobacco environments, and truckers’ related patterns and practices were synthesized in this review to inform needed opportunities for high-impact interventions that target factors beyond individual health knowledge and skills (Ng et al., 2015, Frieden, 2010, Crizzle et al., 2017). Only four reviewed studies used environmental measures (Apostolopoulos et al., 2012, Apostolopoulos et al., 2011, Lincoln et al., 2018, McGuirt et al., 2019) to examine trucking settings and only one study used validated environmental tools (McGuirt et al., 2019). A higher number of studies explored perceptions about environmental factors that influence health patterns and practices (Apostolopoulos et al., 2013, Hege et al., 2019, Holmes and Power, 1996, Layne et al., 2009, Lemke et al., 2016, McDonough et al., 2014, Passey et al., 2014, Staško and Neale, 2007, Turner and Reed, 2011, Versteeg et al., 2018, Wenger, 2008, Whitfield Jacobson et al., 2007); however, these perceptions did not consider truckers’ views of possible environmental change strategies to improve health. Last, there was no evidence about trucking tobacco environments. Thus, a main contribution of this review is a highlighted dearth of empirical information about attributes of trucking environments requiring intervention that negatively influence truckers’ reported food, PA, and tobacco patterns and practices.

While not directly related to the specific focus of our review, several reviewed research studies described an unsupportive trucking workplace culture that was at times implicated for food, PA, and/or tobacco practices (Apostolopoulos et al., 2013, Apostolopoulos et al., 2016, Apostolopoulos et al., 2011, Lemke et al., 2016, McDonough et al., 2014, Wenger, 2008, Johnson et al., 2021, Shattell et al., 2012, Williams et al., 2017). In general, the structure and nature (sedentary, stressful, remote) of the job as well as characteristics of trucking settings (limited health care) were reported barriers to positive health patterns and practices. This indicates future steps to build the research base and inform policy, systems, and environmental change efforts to improve truckers’ workplace environments also need to account for truckers’ views and socio-cultural contexts and “begin with the end in mind” by understanding the acceptability, appropriateness, and feasibility of potential built environment interventions (Weiner et al., 2017, Klesges et al., 2005, Proctor et al., 2011). Specific recommendations to move the state of the science forward are described in more detail below.

### 3.4 Future directions

#### 3.4.1 Trucking food environments

Both objective and perceived assessments of the food environment are widely applied in the public health nutrition literature (Neve et al., 2021, National Collaborative on Childhood Obesity Research, 2019) and are promising approaches to improve the state of the science regarding trucking food environments. For example, details about the development and reliability and validity of the HEATWAI tool used to measure food and PA environments in two studies (Apostolopoulos et al., 2012, Apostolopoulos et al., 2011) and the National Institute for Occupational Safety and Health checklist used in one study (Lincoln et al., 2018) were not able to be identified. To build a coherent evidence base to inform future trucking food environment interventions, the use of standard tools assessed for reliability and validity is critical. As one example, the Nutrition Environments Measures Survey (NEMS) variants (e.g., for convenience stores, restaurants, vending machines) (Center for Health Behavior Research, 2021) are publicly available, accompanied by training, and have standardized scoring protocols for analysis. Additionally, standardized survey metrics for assessing dietary patterns and practices (e.g., Behavioral Risk Factor Surveillance System (diet and alcohol) (Centers for Disease Control and Prevention, 2019), National Cancer Institute Fruit and Vegetable screener (National Cancer Institute, 2021), or the Beverage Intake Questionnaire for Habitual Beverage Intake (Fausnacht et al., 2020) were not consistently applied and are also recommended for trucking research moving forward. Improving the use of standard measures will be especially useful for correlational research linking food environment properties to truckers’ dietary purchases.

#### 3.4.2 Physical activity environments

Measures of the PA environment were also limited. This is unsurprising, as most of the evidence on the association between environment and PA behavior comes from data on individuals’ perceptions of the environment rather than objective assessments (Brownson et al., 2009). However, more information about characteristics of existing environments is needed to guide the selection, adaptation, and implementation of built environment interventions to promote PA. For example, assessing the presence or absence and quality of features that affect PA (e.g., streets, sidewalks, public spaces, signage, litter, lighting, incivilities, shade) (Brownson et al., 2009) can identify barriers and aid in the selection of built environment interventions to improve PA patterns and practices among trucking settings. Moving forward, the Workplace Walkability Audit could be adapted and pilot tested for various trucking settings to assess PA attributes (Dannenberg et al., 2005).

Comparing PA patterns and practices across studies to inform PA interventions was also difficult due to the different measures used in each study. Only two studies (Ronna et al., 2016, Turner and Reed, 2011) that assessed PA used measures aligned with meeting the national PA guidelines (i.e., 150 min per week of moderate-intensity aerobic activity or 75 min of vigorous intensity aerobic activity or an equivalent combination of each, and two sessions of full-body strength training per week) (HHS, 2018). For example, one study assessed whether truckers engaged in 30 min of “sustained” PA three times per week (Whitfield Jacobson et al., 2007). This is difficult to align with national PA recommendations, as aerobic activity does not need to be done in 30-minute periods. The most recent version of the PA guidelines specifies that any duration counts, while the 2008 version specified that aerobic activity needed to be done 10 minutes or more at a time (HHS, 2018). Additionally, only two studies fully assessed compliance with PA guidelines, including the strength training portion of the recommendations. Two studies assessed frequency of stretching (Mullins et al., 2013, Turner and Reed, 2011), which is not included in the guidelines and is not associated with chronic disease prevention (HHS, 2018).

Last, in 1998 a reporter for *Overdrive* noted truck stop gyms were “popping up around the country” to address truck drivers’ health (Cox, 1998). Truckers in some of the reviewed research described truck stop gyms (when available) as “ratty” or time a barrier to their use (Lemke et al., 2016). Evidence-based interventions, such as built environment changes to facilitate PA (Kahn et al., 2002, McCormack and Shiell, 2011, Sallis et al., 2012), may fail to be effective initially and over time if end-users’ perceptions are not considered (Weiner et al., 2017, Klesges et al., 2005, Proctor et al., 2011). Truckers’ buy-in will be critical for future interventions.

#### 3.4.3 Tobacco

No studies to date have examined tobacco environments (either objective or perceived) for truckers (e.g., tobacco availability and marketing in truck stops). The lack of objective and perceived environmental data on retail tobacco environments for truckers represents a considerable gap in the literature given the high prevalence of smoking reported among truckers (Angeles et al., 2014, Hege et al., 2019, Korelitz et al., 1993, Olson et al., 2016, Olson et al., 2016, Crizzle et al., 2020, Ronna et al., 2016, Sieber et al., 2014, Staško and Neale, 2007). Methods to assess the retail tobacco marketing environment have been developed (Lee et al., 2014, Henriksen et al., 2016). Given the high prevalence of smoking and the frequency with which truckers patronize truck stops, these settings may be an ideal location to identify opportunities to support policy and environmental changes conducive to smoking cessation. Globally, point-of-sale tobacco display bans have been found to reduce adult daily smoking by approximately 7% (Ribisl et al., 2017) and a menthol sales ban was found to reduce retail sales by 93% in Ontario (Page et al., 2020). Additionally, a 2019 study by Rust et al. found stores authorized by the Supplemental Nutrition Assistance Program (SNAP) – a federal nutrition assistance program to promote food security among Americans with lower incomes (Tiehen, 2020) – had almost 3 times greater odds of interior tobacco marketing than comparison stores (Rust et al., 2019). Of note, many truck stop locations are SNAP-authorized (USDA, 2021), indicating these sites may have more tobacco advertisements than other locations and thus may be prime sites for feasible policy solutions. Similar environmental approaches to identify smoking cessation intervention opportunities may also increase gender equality, given that women truckers have substantially higher rates of smoking relative to their male counterparts (Birdsey et al., 2015, Layne et al., 2009).

### 3.5 Health disparities, health equity, and the COVID-19 pandemic

More information about trucking population differences in access to and experiences with trucking food, PA, and tobacco environments is needed by sociodemographic characteristics, given this was a limited focus among reviewed studies. The trucking workforce is diversifying (Cheesman Day and Hait, 2019) and health inequities experienced by truckers in general (Sieber et al., 2014) are likely more severe for women and racial and ethnic minority groups in this occupation (Singh et al., 2017). Understanding how trucking populations navigate their environments is critical, including experiences such as safety concerns (Gray and Lindsay, 2019) which may disproportionately affect certain populations and influence how truckers interact with truck stop food, PA, or tobacco amenities. Also, review results may not be reflective of the current trucking food, PA, or tobacco environment. Since the COVID-19 pandemic truckers, who are essential workers, have reported lower pay and closed travel stops and restaurants (or, in some cases, a shift to drive-through ordering) (Hennessy-Fiske, 2020, Cole, 2021, Dills, 2021, Maiden, 2020), which suggests opportunities for healthy eating and PA while on the road may be further reduced.

### 3.6 Limitations

This review has several important contributions: 1) the review scope is a novel characterization of trucking environments and truckers’ patterns and practices related to food, PA, and tobacco that have not been investigated previously; 2) review results demonstrate the state of the science regarding trucking food, PA, and tobacco environments is underdeveloped and requires more focus using better measures; and 3) several future directions are highlighted that introduce public health concepts that could be applied to complement and build on the work of occupational health and safety scientists to improve the state of the science in this regard. However, review results should be interpreted with respect to limitations. Results are limited by the search databases used, which may have been inadequate to identify all articles meeting the inclusion criteria. Five research databases, Google searches, and reference searching were used to minimize this limitation. Some articles identified for review inclusion had a specific field focus (i.e., mental health), so relevant articles may have been overlooked during the title/abstract or reference searching processes due to a lack of perceived focus on food, PA, or tobacco. However, researchers were liberal in flagging sources for full-text review. The exclusion of gray literature is also a limitation given industry reports may have included important information but were not included. Given a source quality assessment was a priority, gray literature was outside the scope of this work. Last, results in majority are reflective of U.S. trucking settings and more investigations are warranted in Canada.

## 4 Conclusion

The science of trucking food, PA, and tobacco environments is underdeveloped and requires much more focus using validated measures. Future steps to build the research base and inform policy, systems, and environmental change efforts to improve truckers’ workplace environments should account for truckers’ views and socio-cultural contexts and “begin with the end in mind” by understanding the acceptability, appropriateness, and feasibility of potential built environment interventions aimed at improving truckers’ food, PA, and tobacco patterns and practices.

## 5 Declarations

### 5.1 Ethics approval and consent to participate

Not applicable; Review did not engage human subjects.

### 5.2 Consent for publication

Not applicable.

### 5.3 Availability of data and materials

Review data is available in reported Tables. The search strategy and quality review indices are available in supplementary files.

## Authors contributions

BH led all aspects of this review. BH, LB, LM, RM, and CBS were responsible for research conception and review design. BH and KK implemented the search strategy and extracted/reviewed data in collaboration with LB, LM, and CBS. BH and LB completed the quality assessment. BH, LB, and LM were responsible for writing the manuscript and KK assisted with Tables and formatted the manuscript to Journal requirements. All authors critically reviewed the draft manuscript and approved the final version.

## Funding

This research was supported by the Louisiana State University Agricultural Center and the U.S. Department of Agriculture National Institute of Food and Agriculture, Hatch project 1024670.

## Declaration of Competing Interest

The authors declare that they have no known competing financial interests or personal relationships that could have appeared to influence the work reported in this paper.
