# Supplementary material for: A systematic review of trucking food, physical activity, and tobacco environments and tractor-trailer drivers’ related patterns and practices in the United States and Canada, 1993–2021
Source: Prev Med Rep. 2022 Mar 8;26:101760. doi: 10.1016/j.pmedr.2022.101760 (PMC8924679; doi:10.1016/j.pmedr.2022.101760)
Supplement: Supplementary Data 1 [file mmc1.docx]

**Supplement: Search Databases and Key Term Application**

MEDLINE

Truck* OR Trucker OR “long-haul” OR "long-haul truck drivers" OR “long-haul truckers” OR “tractor-trailer” OR “professional driver*” OR “motor freight driver" OR “long-haul commercial truck driver”**(TX All Text)**

**AND**

"United States" OR "North America" OR "United States of America" OR Alabama OR Alaska OR  Arizona OR  Arkansas OR California OR  Colorado OR  Connecticut OR Delaware OR  Florida OR  Georgia OR  Hawaii OR  Idaho OR  Illinois OR Indiana OR Iowa OR  Kansas OR  Kentucky  OR Louisiana OR  Maine OR  Maryland OR  Massachusetts OR Michigan OR  Minnesota OR  Mississippi OR Missouri OR Montana OR Nebraska OR Nevada OR “New Hampshire” OR “New Jersey” OR “New Mexico” OR “New York” OR “North Carolina” OR “North Dakota” OR  Ohio OR  Oklahoma OR  Oregon OR  Pennsylvania OR “Rhode Island” OR  “South Carolina” OR  “South Dakota” OR Tennessee OR  Texas OR Utah OR  Vermont OR Virginia OR  Washington OR  “West Virginia” OR  Wisconsin OR  Wyoming  OR Canada OR Yukon OR “Northwest Territories” OR Nunavut OR “British Columbia” OR Alberta OR Saskatchewan OR Manitoba OR Ontario OR Quebec OR “Newfoundland Labrador” OR “New Brunswick” OR “Nova Scotia” OR “Prince Edward Island” **(GN Geographic Subject Minor)**

**AND**

behavior* OR “behavior change” OR "choice behavior*" OR “health behavior*” OR “health resource*” OR attitude* OR belief* OR barrier* OR beverage* OR diet* OR “dietary behavior” OR exercise* OR food* OR “food choice*” OR health* OR “lifestyle activit*” OR nutrition* OR “physical activity” OR “physical activities” OR  “physical fitness” OR "health disparities*" or “healthful” OR tobacco or nicotine or cigarette* or vaping or vape or ecigarette or E-Cigarette or "electric cigarette" **(TX All Text)**

**AND**

“built environment” OR “rest area*” OR “rest stop*” OR transportation* OR “truck cab” OR “truck stop*” OR “truck terminal*” OR "trucking work environment" OR warehouse OR workplace* OR “work setting*” OR “work site” OR “work environment” OR Restaurant* or "work organization" or "workplace"**(TX All Text)**

PubMed

Truck* OR Trucker OR “long-haul” OR "long-haul truck drivers" OR “long-haul truckers” OR “tractor-trailer” OR “professional driver*” OR “motor freight driver" OR “long-haul commercial truck driver”**(Title/Abstract)**

**AND**

"United States" or "North America" or "United States of America" or Alabama OR Alaska OR  Arizona OR  Arkansas OR California OR  Colorado OR  Connecticut OR Delaware OR  Florida OR  Georgia OR  Hawaii OR  Idaho OR  Illinois OR Indiana OR Iowa OR  Kansas OR  Kentucky  OR Louisiana OR  Maine OR  Maryland OR  Massachusetts OR Michigan OR  Minnesota OR  Mississippi OR Missouri OR Montana OR Nebraska OR Nevada OR “New Hampshire” OR “New Jersey” OR “New Mexico” OR “New York” OR “North Carolina” OR “North Dakota” OR  Ohio OR  Oklahoma OR  Oregon OR  Pennsylvania OR “Rhode Island” OR  “South Carolina” OR  “South Dakota” OR Tennessee OR  Texas OR Utah OR  Vermont OR Virginia OR  Washington OR  “West Virginia” OR  Wisconsin OR  Wyoming  OR Canada OR Yukon OR “Northwest Territories” OR Nunavut OR “British Columbia” OR Alberta OR Saskatchewan OR Manitoba OR Ontario OR Quebec OR “Newfoundland Labrador” OR “New Brunswick” OR “Nova Scotia” OR “Prince Edward Island” **(Title/Abstract)**

**AND**

behavior* OR “behavior change” OR "choice behavior*" OR “health behavior*” OR “health resource*” OR attitude* OR belief* OR barrier* OR beverage* OR diet* OR “dietary behavior” OR exercise* OR food* OR “food choice*” OR health* OR “lifestyle activit*” OR nutrition* OR “physical activity” OR “physical activities” OR  “physical fitness” OR "health disparities*" or “healthful” OR tobacco or nicotine or cigarette* or vaping or vape or ecigarette or E-Cigarette or "electric cigarette" **(Title/Abstract)**

**AND**

“built environment” OR “rest area*” OR “rest stop*” OR transportation* OR “truck cab” OR “truck stop*” OR “truck terminal*” OR "trucking work environment" OR warehouse OR workplace* OR “work setting*” OR “work site” OR “work environment” OR Restaurant* or "work organization" or "workplace"**(Title/Abstract)**

Web of Science

Truck* OR Trucker OR “long-haul” OR "long-haul truck drivers" OR “long-haul truckers” OR “tractor-trailer” OR “professional driver*” OR “motor freight driver" OR “long-haul commercial truck driver”**(Topic)**

**AND**

"United States" or "North America" or "United States of America" or Alabama OR Alaska OR  Arizona OR  Arkansas OR California OR  Colorado OR  Connecticut OR Delaware OR  Florida OR  Georgia OR  Hawaii OR  Idaho OR  Illinois OR Indiana OR Iowa OR  Kansas OR  Kentucky  OR Louisiana OR  Maine OR  Maryland OR  Massachusetts OR Michigan OR  Minnesota OR  Mississippi OR Missouri OR Montana OR Nebraska OR Nevada OR “New Hampshire” OR “New Jersey” OR “New Mexico” OR “New York” OR “North Carolina” OR “North Dakota” OR  Ohio OR  Oklahoma OR  Oregon OR  Pennsylvania OR “Rhode Island” OR  “South Carolina” OR  “South Dakota” OR Tennessee OR  Texas OR Utah OR  Vermont OR Virginia OR  Washington OR  “West Virginia” OR  Wisconsin OR  Wyoming  OR Canada OR Yukon OR “Northwest Territories” OR Nunavut OR “British Columbia” OR Alberta OR Saskatchewan OR Manitoba OR Ontario OR Quebec OR “Newfoundland Labrador” OR “New Brunswick” OR “Nova Scotia” OR “Prince Edward Island” **(Topic)**

**AND**

behavior* OR “behavior change” OR "choice behavior*" OR “health behavior*” OR “health resource*” OR attitude* OR belief* OR barrier* OR beverage* OR diet* OR “dietary behavior” OR exercise* OR food* OR “food choice*” OR health* OR “lifestyle activit*” OR nutrition* OR “physical activity” OR “physical activities” OR  “physical fitness” OR "health disparities*" or “healthful” OR tobacco or nicotine or cigarette* or vaping or vape or ecigarette or E-Cigarette or "electric cigarette" **(Topic)**

**AND**

“built environment” OR “rest area*” OR “rest stop*” OR transportation* OR “truck cab” OR “truck stop*” OR “truck terminal*” OR "trucking work environment" OR warehouse OR workplace* OR “work setting*” OR “work site” OR “work environment” OR Restaurant* or "work organization" or "workplace"**(Topic)**

CINHAL

Truck* OR Trucker OR “long-haul” OR "long-haul truck drivers" OR “long-haul truckers” OR “tractor-trailer” OR “professional driver*” OR “motor freight driver" OR “long-haul commercial truck driver”**(AB Abstract)**

**AND**

"United States" or "North America" or "United States of America" or Alabama OR Alaska OR  Arizona OR  Arkansas OR California OR  Colorado OR  Connecticut OR Delaware OR  Florida OR  Georgia OR  Hawaii OR  Idaho OR  Illinois OR Indiana OR Iowa OR  Kansas OR  Kentucky  OR Louisiana OR  Maine OR  Maryland OR  Massachusetts OR Michigan OR  Minnesota OR  Mississippi OR Missouri OR Montana OR Nebraska OR Nevada OR “New Hampshire” OR “New Jersey” OR “New Mexico” OR “New York” OR “North Carolina” OR “North Dakota” OR  Ohio OR  Oklahoma OR  Oregon OR  Pennsylvania OR “Rhode Island” OR  “South Carolina” OR  “South Dakota” OR Tennessee OR  Texas OR Utah OR  Vermont OR Virginia OR  Washington OR  “West Virginia” OR  Wisconsin OR  Wyoming  OR Canada OR Yukon OR “Northwest Territories” OR Nunavut OR “British Columbia” OR Alberta OR Saskatchewan OR Manitoba OR Ontario OR Quebec OR “Newfoundland Labrador” OR “New Brunswick” OR “Nova Scotia” OR “Prince Edward Island” **(AB Abstract)**

**AND**

behavior* OR “behavior change” OR "choice behavior*" OR “health behavior*” OR “health resource*” OR attitude* OR belief* OR barrier* OR beverage* OR diet* OR “dietary behavior” OR exercise* OR food* OR “food choice*” OR health* OR “lifestyle activit*” OR nutrition* OR “physical activity” OR “physical activities” OR  “physical fitness” OR "health disparities*" or “healthful” OR tobacco or nicotine or cigarette* or vaping or vape or ecigarette or E-Cigarette or "electric cigarette" **(AB Abstract)**

**AND**

“built environment” OR “rest area*” OR “rest stop*” OR transportation* OR “truck cab” OR “truck stop*” OR “truck terminal*” OR "trucking work environment" OR warehouse OR workplace* OR “work setting*” OR “work site” OR “work environment” OR Restaurant* or "work organization" or "workplace"**(AB Abstract)**

APA PsycInfo

Truck* OR Trucker OR “long-haul” OR "long-haul truck drivers" OR “long-haul truckers” OR “tractor-trailer” OR “professional driver*” OR “motor freight driver" OR “long-haul commercial truck driver”**(TX All Text)**

**AND**

"United States" or "North America" or "United States of America" or Alabama OR Alaska OR  Arizona OR  Arkansas OR California OR  Colorado OR  Connecticut OR Delaware OR  Florida OR  Georgia OR  Hawaii OR  Idaho OR  Illinois OR Indiana OR Iowa OR  Kansas OR  Kentucky  OR Louisiana OR  Maine OR  Maryland OR  Massachusetts OR Michigan OR  Minnesota OR  Mississippi OR Missouri OR Montana OR Nebraska OR Nevada OR “New Hampshire” OR “New Jersey” OR “New Mexico” OR “New York” OR “North Carolina” OR “North Dakota” OR  Ohio OR  Oklahoma OR  Oregon OR  Pennsylvania OR “Rhode Island” OR  “South Carolina” OR  “South Dakota” OR Tennessee OR  Texas OR Utah OR  Vermont OR Virginia OR  Washington OR  “West Virginia” OR  Wisconsin OR  Wyoming  OR Canada OR Yukon OR “Northwest Territories” OR Nunavut OR “British Columbia” OR Alberta OR Saskatchewan OR Manitoba OR Ontario OR Quebec OR “Newfoundland Labrador” OR “New Brunswick” OR “Nova Scotia” OR “Prince Edward Island” **(GN Geographic Subject Minor)**

**AND**

behavior* OR “behavior change” OR "choice behavior*" OR “health behavior*” OR “health resource*” OR attitude* OR belief* OR barrier* OR beverage* OR diet* OR “dietary behavior” OR exercise* OR food* OR “food choice*” OR health* OR “lifestyle activit*” OR nutrition* OR “physical activity” OR “physical activities” OR  “physical fitness” OR "health disparities*" or “healthful” OR tobacco or nicotine or cigarette* or vaping or vape or ecigarette or E-Cigarette or "electric cigarette" **(TX All Text)**

**AND**

“built environment” OR “rest area*” OR “rest stop*” OR transportation* OR “truck cab” OR “truck stop*” OR “truck terminal*” OR "trucking work environment" OR warehouse OR workplace* OR “work setting*” OR “work site” OR “work environment” OR Restaurant* or "work organization" or "workplace"**(TX All Text)**
