# Supplementary material for: A systematic review of trucking food, physical activity, and tobacco environments and tractor-trailer drivers’ related patterns and practices in the United States and Canada, 1993–2021
Source: Prev Med Rep. 2022 Mar 8;26:101760. doi: 10.1016/j.pmedr.2022.101760 (PMC8924679; doi:10.1016/j.pmedr.2022.101760)
Supplement: Supplementary Data 2 [file mmc2.docx]

| Supplemental Table. 2018 Mixed Method Appraisal Tool Scores for Included Research in a Systematic Review of Trucking Food, Physical Activity, and Tobacco Environments and Truckers’ Related Patterns and Practices in the United States and Canada (n=38). | | | | | | | |
| --- | --- | --- | --- | --- | --- | --- | --- |
| **Quantitative Descriptive Studies**  **Author, Year** | **Are there clear research questions?** | **Do the collected data allow to address the research questions?** | **Is the sampling strategy relevant to address the research question?** | **Is the sample representative of the target population?** | **Are the measurements appropriate?** | **Is the risk of nonresponse bias low?** | **Is the statistical analysis appropriate to answer the research question?** |
| Angeles, 2014^1^ | Yes | Yes | Can’t tell. Why: sample size calculated for 13 of 20 trucking companies in SW Ontario. No information is provided about why these companies were selected | No. Why: sample size is calculated for 13 of 20 trucking companies in SW Ontario | Yes | No. Why: Response rate 49% | Yes |
| Apostolopoulos, 2011^2^ | Yes | No. Why: data describes the environment but does not assess relationship | Can’t tell. Why: vague information given about selecting sites that represent varied geographic, corporate, etc. settings | No. Why: See columns to the left | Can’t Tell. Why: Details about the creation, validity, and pilot testing of this tool are not described and cannot be found | Can’t tell. Why: No details provided about data collection or if site management declined tool implementation | No. Why: data describes the environment but does not assess relationship |
| Apostolopoulos,2012^3^ | Yes | No. Why: data describes the environment but does not assess relationship | Can’t tell. Why: vague information given about selecting sites that represent geographic, corporate, etc. settings | No. Why: does not seem representative of all truck stops in study setting (see column to the left) | Can’t tell. Why: Details about the creation, validity, and pilot testing of this tool are not described and cannot be found | Can’t tell. Why: vague information given about selecting sites that represent geographic, corporate, etc. settings and/or if sites declined measurement | No. Why: data describes the environment but does not assess relationship |
| Apostolopoulos, 2013^4^ | Yes | No. Why: data describes health status and access but does not assess relationship | No. Why: no details about why recruitment sites were chosen or eligibility criteria | No. Why: women are excluded | Yes | Can’t tell. Why: no information given about those who declined participation | No. Why: data describes the truckers’ health and work characteristics but does not assess ‘links’ |
| Apostolopoulos, 2016^5^ | Yes | Yes | Can’t tell. Why: vague information given about selecting sites that represent varied geographic, corporate, etc. settings | No. Why: convenience sampling | Can’t tell. Why: Details about the creation, validity, and pilot testing of this tool are not described and cannot be found | Can’t tell. Why: no information about if sites declined measurement | Yes |
| Bachmann, 2018^6^ | Yes | Yes | No. Why: not enough information about site selection | No. Why: convenience sampling | Yes | No. Why: response rate 65.55% | No. Why: Study aims are descriptive, and analysis is inferential |
| Birdsey, 2015^7^ | Yes | Yes | Yes | Yes | Yes | Yes | Yes |
| Crizzle, 2020^8^ | Yes | Yes | Can’t tell. Why: not enough information about site selection or survey recruitment | Can’t tell. Why: no information on how sites were selected or how drivers were approached | Yes | Can’t tell. Why: no information on non-participants | Yes |
| Heaton, 2015^9^ | Yes | Yes | Yes | Can’t tell. Why: not enough information to determine | Can’t tell. Why: not enough information to determine | Yes | Can’t Tell. No details provided for why repeated measures ANOVA was the best choice for data analysis |
| Hege, 2019^10^ | Yes | Yes | Yes | No. Why: convenience sampling | No. Why: non-standardized measures used | Can’t tell. Why: not enough information to determine | Yes |
| Holmes, 1996^11^ | Yes | Yes | Can’t tell. Why: selection of employee participants not described | Can’t tell. Why: selection of employee participants not described | Yes | Can’t tell. Why: no information on non-participants | Yes |
| Korelitz, 1993^12^ | Yes | Yes | Yes | No. Why: convenience sampling | Can’t tell. Why: Some measures used are non-standardized | No. Why: no information on non-participants | No. Why: Study aims are descriptive, and analysis is inferential |
| Layne, 2009^13^ | Yes | Yes | Yes | No. Why: convenience sampling | Yes | Can’t tell. Why: no information details about non-participants | Yes |
| Lincoln, 2018^14^ | Yes | Yes | Yes | No. Why: sampling strategy cannot be used to generate national estimates for truck stop amenities | Can’t tell. Why: Details about the creation, validity, and pilot testing of this tool are not described and cannot be found | No. Why: no information about if sites declined measurement | Yes |
| McGuirt, 2019^15^ | Yes | Yes | Yes | No. Why: Only toll-free routes | Yes | Yes | Yes |
| Mullins, 2013^16^ | Yes | Yes | Yes | No. Why: convenience sampling | Yes | Can’t tell. Why: no information details about non-participants | Yes |
| Olson, 2009^17^ | Yes | Yes | Yes | No. Why: convenience sampling | Yes | Yes | Yes |
| Olson, 2016^18^ | Yes | Yes | Yes | No. Why: convenience sampling | Yes | Yes | Yes |
| Olson, 2017^19^ | Yes | Yes | Yes | No. Why: convenience sampling | Yes | No. Why: response rate 75% | Yes |
| Ronna, 2016^20^ | Yes | Yes | Yes | No. Why: convenience sampling | Yes | Can’t tell: Why: not enough information on non-participants | Yes |
| Shattell, 2012^21^ | Yes | Yes | Yes | No. Why: Collected data from truckers at one truck stop and details about why this location was chosen are not provided | Can’t Tell. Why: Adapted survey with no details provided on reliability/validity testing of adapted version | Can’t tell: Why: not enough information on non-participants | Yes |
| Solomon, 2004^22^ | Yes | Yes | Yes | No. Why: convenience sampling | Can’t tell. Why: no information on measures | No. Why: low response rates | Yes |
| Sieber, 2014^23^ | Yes | Yes | Yes | Yes | Yes | Yes | Yes |
| Thiese, 2015^24^ | Yes | Yes | Yes | No. Why: convenience sampling | No. Why: did not use standardized self-report measures | Can’t tell. Why: not enough information on non-participants | Yes |
| Turner, 2011^25^ | Yes | Yes | Yes | No. Why: convenience sampling | No. Why: did not use standardized self-report measures | Can’t tell. Why: not enough information on non-participants | No. Why: Study aims are exploratory and analysis is predictive |
| Van Hemel, 1998^26^ | Yes | Yes | Yes | No. Why: convenience sampling | Can’t Tell. Why: limited information about where measures are sourced from | No. Why: response rate 20% | No. Why: no theory driving the use of inferential tests |
| Whitfield Jacobson, 2007^27^ | Yes | Yes | Yes | No. Why: convenience sampling | Yes | Can’t tell. Why: cannot find details about response rate | Yes |
| **Qualitative Studies**  **Author, Year** | **Are there clear research questions?** | **Do the collected data allow to address the research questions?** | **Is the qualitative approach appropriate to answer the research question?** | **Are the qualitative data collection methods adequate to address the research question?** | **Are the findings adequately derived from the data?** | **Is the interpretation of results sufficiently substantiated by data?** | **Is there coherence between qualitative data sources, collection, analysis and interpretation?** |
| Johnson, 2021^28^ | Yes | Yes | Yes | Yes | Yes | Yes | Yes |
| Lemke, 2016^29^ | Yes | Yes | Yes | Yes | Yes | Yes | Yes |
| McDonough, 2014^30^ | Yes | Yes | Yes | Yes | Yes | Yes | Yes |
| Passey, 2014^31^ | Yes | Yes | Yes | Yes | Yes | Yes | Yes |
| Shattell, 2010^32^ | Can’t tell. Why: The purpose is to report “findings on the occupational stressors and the mental health of truckers” from a larger study and it is unclear what the research questions are | Can’t tell. Why: The purpose is to report “findings on the occupational stressors and the mental health of truckers” from a larger study and it is unclear what the research questions are | Can’t tell. Why: Research questions are unclear | Yes | Yes | Yes | Can’t tell. Why: Research questions are unclear |
| Wenger, 2008^33^ | No. Why: no study methods described | No. Why: no study methods described | No. Why: no study methods described | No. Why: no study methods described | No. Why: no study methods described | No. Why: very brief conclusion section | No. Why: lack of detail; no methods described |
| Williams, 2017^34^ | Yes | Yes | Yes | Yes | Yes | Yes | Yes |
| **Mixed Method Studies**  **Author, Year** | **Are there clear research questions?** | **Do the collected data allow to address the research questions?** | **Is there an adequate rationale for using a mixed methods design to address the research question?** | **Are the different components of the study effectively integrated to answer the research question?** | **Are the outputs of the integration of qualitative and quantitative components adequately interpreted?** | **Are divergences and inconsistencies between quantitative and qualitative results adequately addressed?** | **Do the different components of the study adhere to the quality criteria of each tradition of the methods involved?** |
| Crizzle, 2020^35^ | Yes | Yes | Yes | Yes | Yes | Yes | Yes |
| Gay Anderson^36^ | Yes | No. Why: unclear how the data corresponded to the purpose statements | No. Why: no rationale is provided | Yes | Can’t tell. Why: vague discussion with limited links to similar work for drawing conclusions | Yes | No. Why: no details on the qualitative methods used. Non-representative quantitative sample with limited details on analysis |
| Staško, 2007^37^ | No. Why: vague explanation of “exploring health care needs and access issues” | Can’t tell. Why: vague explanation of “exploring health care needs and access issues” | Can’t tell. Why: no rationale is provided. | Can’t tell. Why: see columns to the left | Yes | Yes | No. Why: methods and analysis for the qualitative portion are not included. The sample may not be representative |
| Versteeg, 2018^38^ | Yes | Yes | Yes | Yes | Yes | Yes | Yes |

**Supplementary Table References**

1. Angeles R, McDonough B, Howard M, et al. Primary health care needs for a priority population: a survey of professional truck drivers. Work*;* 2014;49(2):175-181.

2. Apostolopoulos Y, Sonmez S, Shattell M, Haldeman L, Strack R, Jones V. Barriers to truck drivers' healthy eating: environmental influences and health promotion strategies. J Workplace Behav Health*.* 2011;26(2):122-143.

3. Apostolopoulos Y, Shattell MM, Sönmez S, Strack R, Haldeman L, Jones V. Active living in the trucking sector: environmental barriers and health promotion strategies. J Phys Act Health*.* 2012;9(2):259-269.

4. Apostolopoulos Y, Soenmez S, Shattell MM, Gonzales C, Fehrenbacher C. Health survey of US long-haul truck drivers: work environment, physical health, and healthcare access. 2013; 46(1):113-123.

5. Apostolopoulos Y, Lemke M, Sönmez S, Hege A. The obesogenic environment of commercial trucking: a worksite environmental audit and implications for systems-based interventions. Am J Health Educ. 2016;47(2):85-93.

6. Bachmann LH, Lichtenstein B, St Lawrence JS, Murray M, Russell GB, Hook EW III. Health risks of American long-distance truckers: results from a multisite assessment. J Occup Environ Med*.* 2018;60(7):E349-E355.

7. Birdsey J, Sieber WK, Chen GX, et al. National survey of US long-haul truck driver health and injury health behaviors. J Occup Environ Med*.* 2015;57(2):210-216.

8. Crizzle AM, McLean M, Malkin J. Risk factors for depressive symptoms in long-haul truck drivers. Int J Environ Res Public Health*.* 2020;17(11).

9. Heaton K, Griffin R. The effects of caffeine use on driving safety among truck drivers who are habitual caffeine users. 2015; 63:333-341.

10. Hege A, Lemke MK, Apostolopoulos Y, Sönmez S. The impact of work organization, job stress, and sleep on the health behaviors and outcomes of U.S. long-haul truck Drivers. Health Educ Behav*.* 2019;46(4):626-36.

11. Holmes SM, Power ML. A Motor Carrier Wellness Program: development and testing. Transp J*.* 1996;35(3):33-48.

12. Korelitz JJ, Fernandez AA, Uyeda VJ, Spivey GH, Browdy BL, Schmidt RT. Health habits and risk factors among truck drivers visiting a health booth during a trucker trade show. Am J Health Promot*.* 1993;8(2):117-23.

13. Layne DM, Rogers B, Randolph SA. Health and gender comparisons in the long-haul trucking industry: a pilot study. AAOHN J. 2009; 57:405-413.

14. Lincoln JE, Birdsey J, Sieber WK, et al. A pilot study of healthy living options at 16 truck stops across the United States. Am J Health Promot. 2018;32(3):546-553.

15. McGuirt JT, Huebner G, Ward R, Jilcott Pitts SB. Food and beverage options at highway rest areas in North Carolina: a mixed-methods audit and geospatial approach. Prevent Chronic Dis. 2019;16:1.

16. Mullins IL, O'Day T, Kan TY. Validation of the health-promoting lifestyle profile II for Hispanic male truck drivers in the Southwest. Clin Nurs Res. 2013; 22:375-394.

17. Olson R, Anger WK, Elliot DL, Wipfli B, Gray M. A new health promotion model for lone workers: results of the Safety & Health Involvement for Truckers (SHIFT) pilot study. J Occup Environ Med*.* 2009;51(11):1233-1246.

18. Olson R, Wipfli B, Thompson SV, et al. Weight Control intervention for truck drivers: the SHIFT randomized controlled trial, United States. Am J Public Health*.* 2016;106(9):1698-1706.

19. Olson R, Thompson SV, Wipfli B, et al. Sleep, dietary, and exercise behavioral clusters among truck drivers with obesity: implications for interventions. J Occup Environ Med. 2016; 58(3), 314-41*.*

20. Ronna BB, Thiese MS, Ott U, et al. The association between cardiovascular disease risk factors and motor vehicle crashes among professional truck drivers. J Occup Environ Med*.* 2016;58(8):828-832.

21. Shattell M, Apostolopoulos Y, Collins C, Sonmez S, Fehrenbacher C. Trucking organization and mental health disorders of truck drivers. Issues Ment Health Nurs*.* 2012;7(33):436-444.

22. Solomon AJ, Doucette JT, Garland E, McGinn T. Healthcare and the long haul: long distance truck drivers - a medically underserved population. Am J Ind Med*.* 2004; 46:463–71

23. Sieber WK, Robinson CF, Birdsey J, et al. Obesity and other risk factors: the national survey of U.S. long-haul truck driver health and injury. Am J Ind Med*.* 2014;57(6):615-26.

24. Thiese MS, Effiong AC, Ott U, et al. A clinical trial on weight loss among truck drivers. Int J Occup Environ Med*.* 2015;6(2):104-112.

25. Turner LM, Reed DB. Exercise among commercial truck drivers. AAOHN J. 2011; 59:429-36.

26. Van Hemel SB, Rogers WC. Survey of truck drivers’ knowledge and beliefs regarding driver fatigue. Transp Res Rec. 1998; 1640(1):65-73.

27. Whitfield Jacobson PJ, Prawitz AD, Lukaszuk JM. Long-haul truck drivers want healthful meal options at truck-stop restaurants. J Am Diet Assoc. 2007;107(12):2125-2129.

28. Johnson JK, Vingilis E, Terry AL. Qualitative study of long-haul truck drivers' health and healthcare experiences. J Occup Environ Med. 2021;63(3):230-237.

29. Lemke MK, Meissen GJ, Apostolopoulos Y. Overcoming barriers in unhealthy settings: a phenomenological study of healthy truck drivers. Glob Qual Nurs Res*.* 2016;3:1-9. DOI: 10.1177/2333393616637023

30. McDonough B, Howard M, Angeles R, et al. Lone workers attitudes towards their health: views of Ontario truck drivers and their managers. BMC Res Notes*.* 2014;7(1):2-15.

31. Passey DG, Robbins R, Hegmann KT, et al. Long haul truck drivers' views on the barriers and facilitators to healthy eating and physical activity. Inter J Workplace Health Manag*.* 2014;7(2):121.

32. Shattell M, Apostolopoulos Y, Sonmez S, Griffin M. Trucking organization and mental health disorders of truck drivers. Issues Ment Health Nurs*.* 2010:436-44.

33. Wenger J. Freedom isn’t free: voices from the truck driving industry. New Solut*.* 2008;18(4):481-491.

34. Williams DF, Thomas SP, Liao-Troth S. The truck driver experience: identifying psychological stressors from the voice of the driver. Transp J. 2017:54-76.

35. Crizzle AM, Toxopeus R, Malkin J. Impact of limited rest areas on truck driver crashes in Saskatchewan: a mixed-methods approach. BMC Public Health*.* 2020;20(1).

36. Anderson G. Determining standards of care for substance abuse and alcohol use in long-haul truck drivers. Nurs Clin North Am. 2008;3(43):357-365.

37. Staško JC, Neale AV. Health care risks and access within the community of Michigan over-the-road truckers. Work. 2007;29(3):205-211.

38. Versteeg K, Amoli T, Cao M, Chin M, Bigelow P, Yazdani A. Mixed-method analysis of truck driver health knowledge using an online forum. Saf Sci. 2018;102:51-59.
